# Supplementary material for: Comparative analysis defines a broader FMRFamide-gated sodium channel family and determinants of neuropeptide sensitivity
Source: J Biol Chem. 2022 May 27;298(7):102086. doi: 10.1016/j.jbc.2022.102086 (PMC9234716; doi:10.1016/j.jbc.2022.102086)
Supplement: Supplemental Figures S1–S4 and Supporting Text [file mmc1.docx]

**Supporting information**

**Comparative analysis defines a broader FMRFamide-gated sodium channel family and determinants of neuropeptide sensitivity**

Mowgli Dandamudi^1^, Harald Hausen^1,2^, Timothy Lynagh^1^*

^1^ Sars International Centre for Marine Molecular Biology, University of Bergen, Norway

^2^ Department of Earth Science, University of Bergen, Norway

*Corresponding author, tim.lynagh@uib.no

**Contents**

**Supporting figuresS2**

Figure S1S2

Figure S2S3

Figure S3S5

Figure S4S6

**Supporting textS7**

Oocyte expression vector and novel cDNA insertsS7

Other cDNA constructsS10

**Supporting referencesS11**

**Supporting figures**


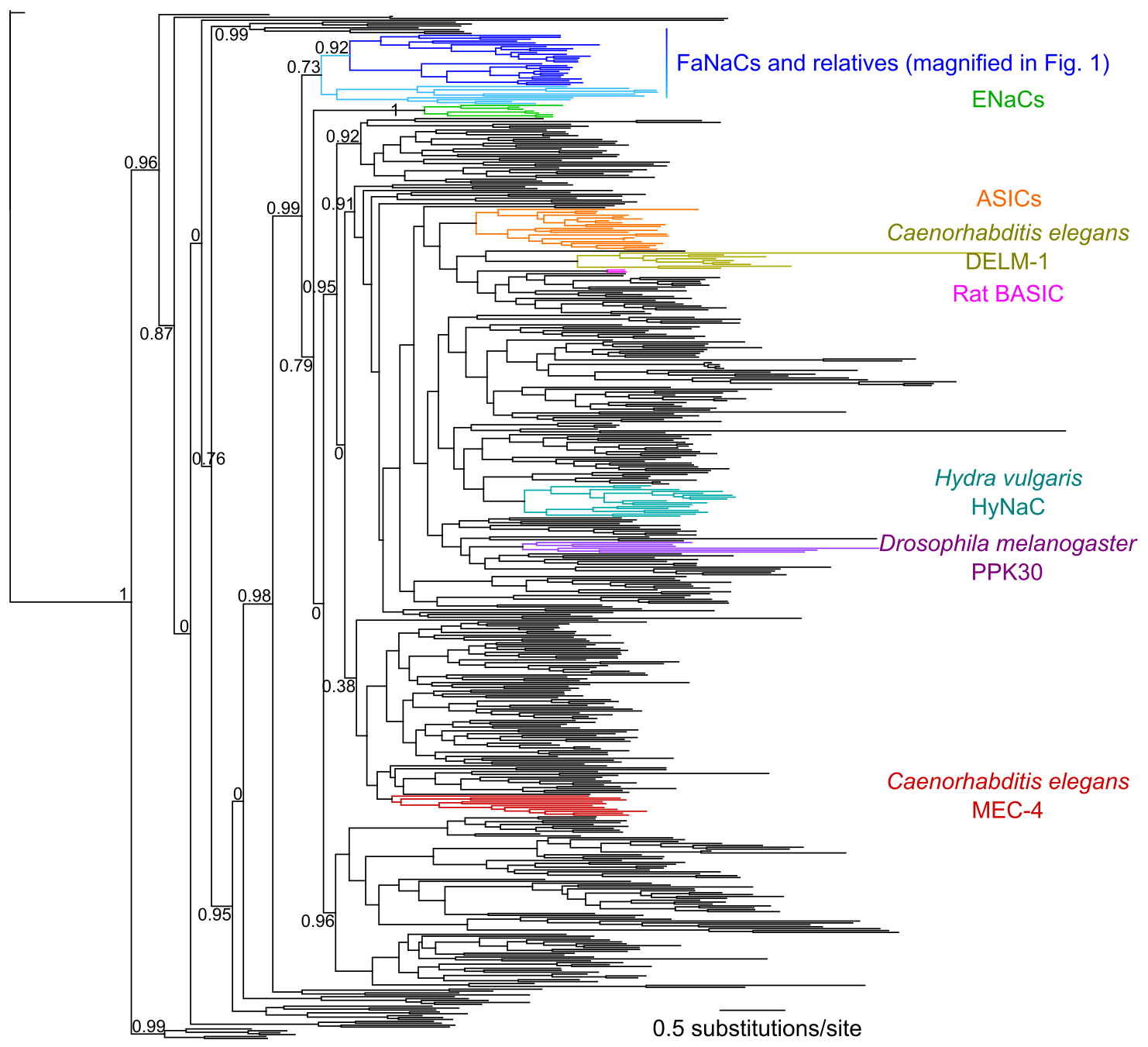


**Figure S1. DEG/ENaC phylogeny.** Unrooted PhyML maximum likelihood tree (ATGC Montpellier Bioinformatics Platform, VT + G calculated as appropriate substitution model) of 544 DEG/ENaC genes from various metazoans. For clarity, only selected branch support values (aLRT SH-like) are shown. Well-known DEG/ENaC sub-families are colored and labeled. Phylogeny is based on MAFFT alignment, both available at https://www.lynaghlab.com/resources.


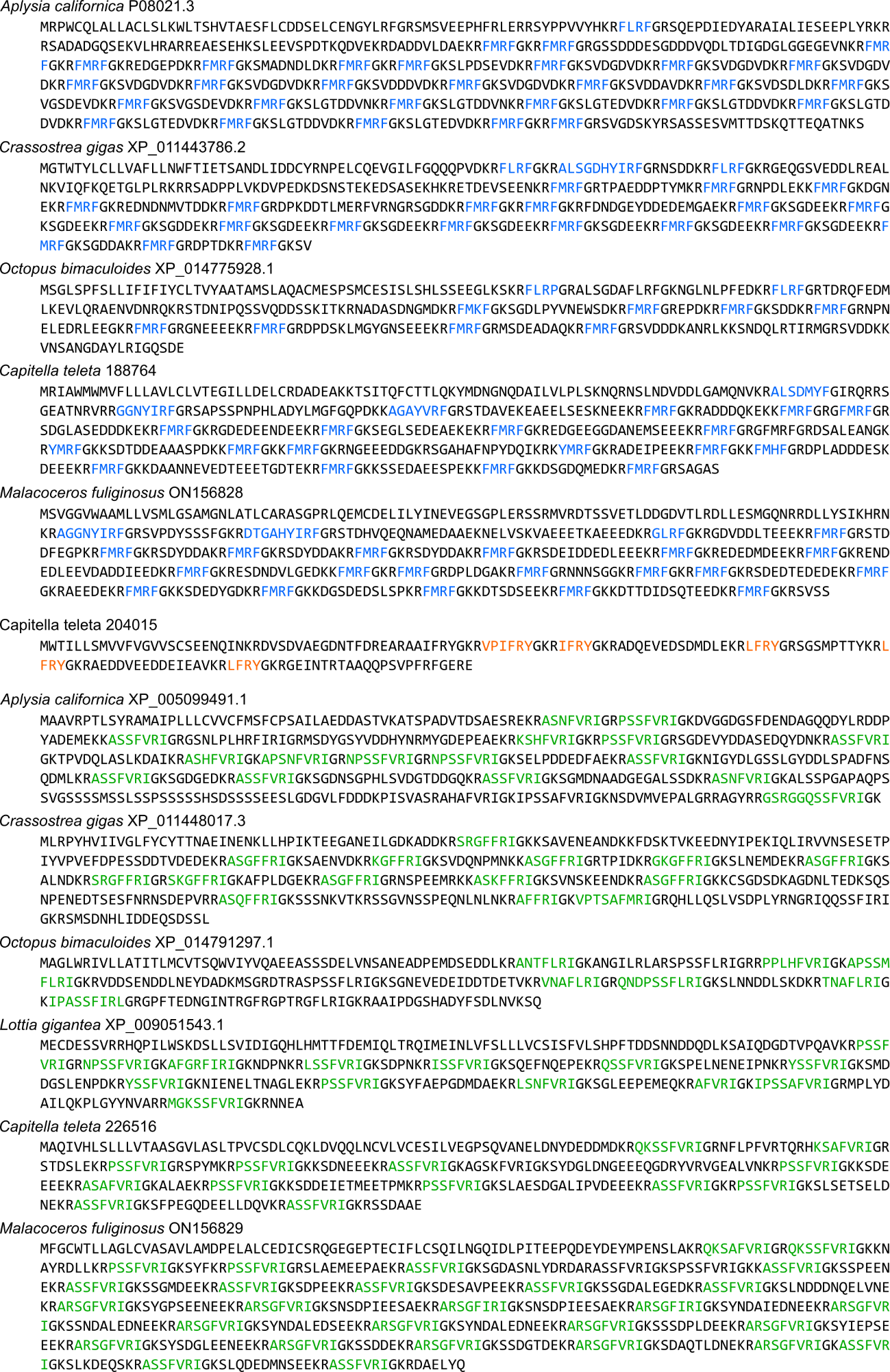


**
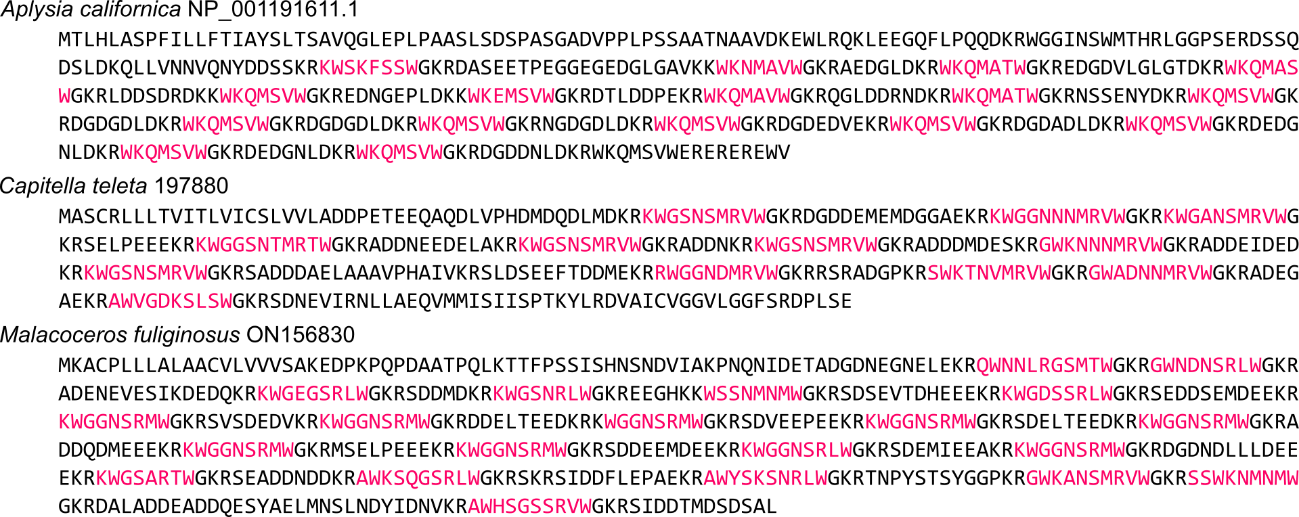
**

**Figure S2. Selected pro-peptides.** Putative neuropeptides, based on double basic residue (e.g. KR) and GR cleavage and amidation sites, are highlighted in color within the pro-peptide: blue, FMRFa; orange, LFRYa; green, FVRIa; magenta, Wamide (or "myoinhibitory peptide"). All sequences/accession codes from NCBI, except for *Capitella teleta*, from Capcal1 genome, Joint Genome Institute, and *Malacoceros fuliginosus*, from our transcriptome resource (42; also available in GenBank via the above accession codes).


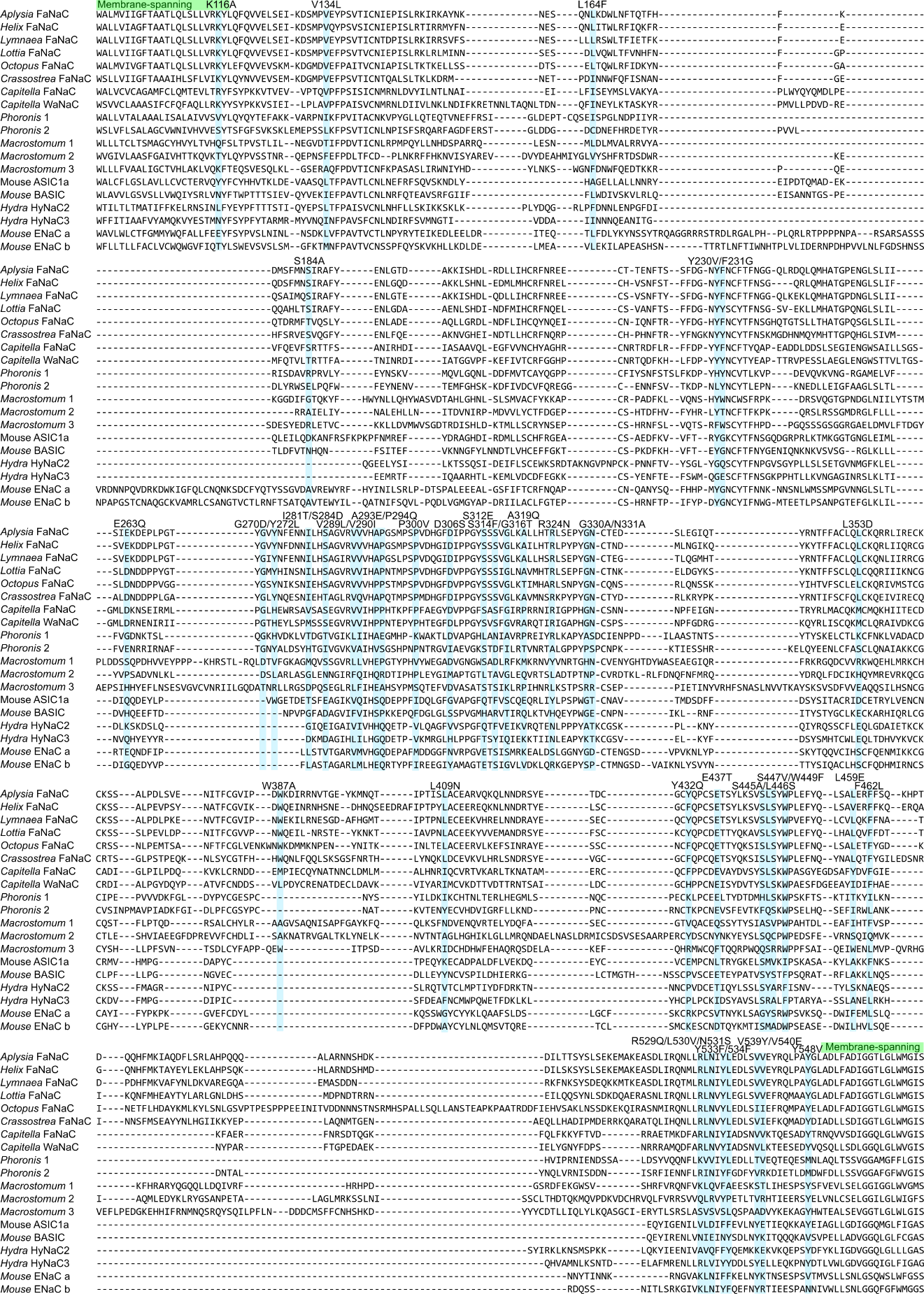


**Figure S3. FaNaC-specific amino acid residues.** Alignment highlighting 43 positions where channels gated by FMRFa possess physico-chemically similar residues and non-FMRFa-gated channels possess different residues. 30 *Aplysia* FaNaC mutants based on these differences are indicated above the alignment. Putative intracellular domains (before and after green membrane-spanning domains) have been excluded.


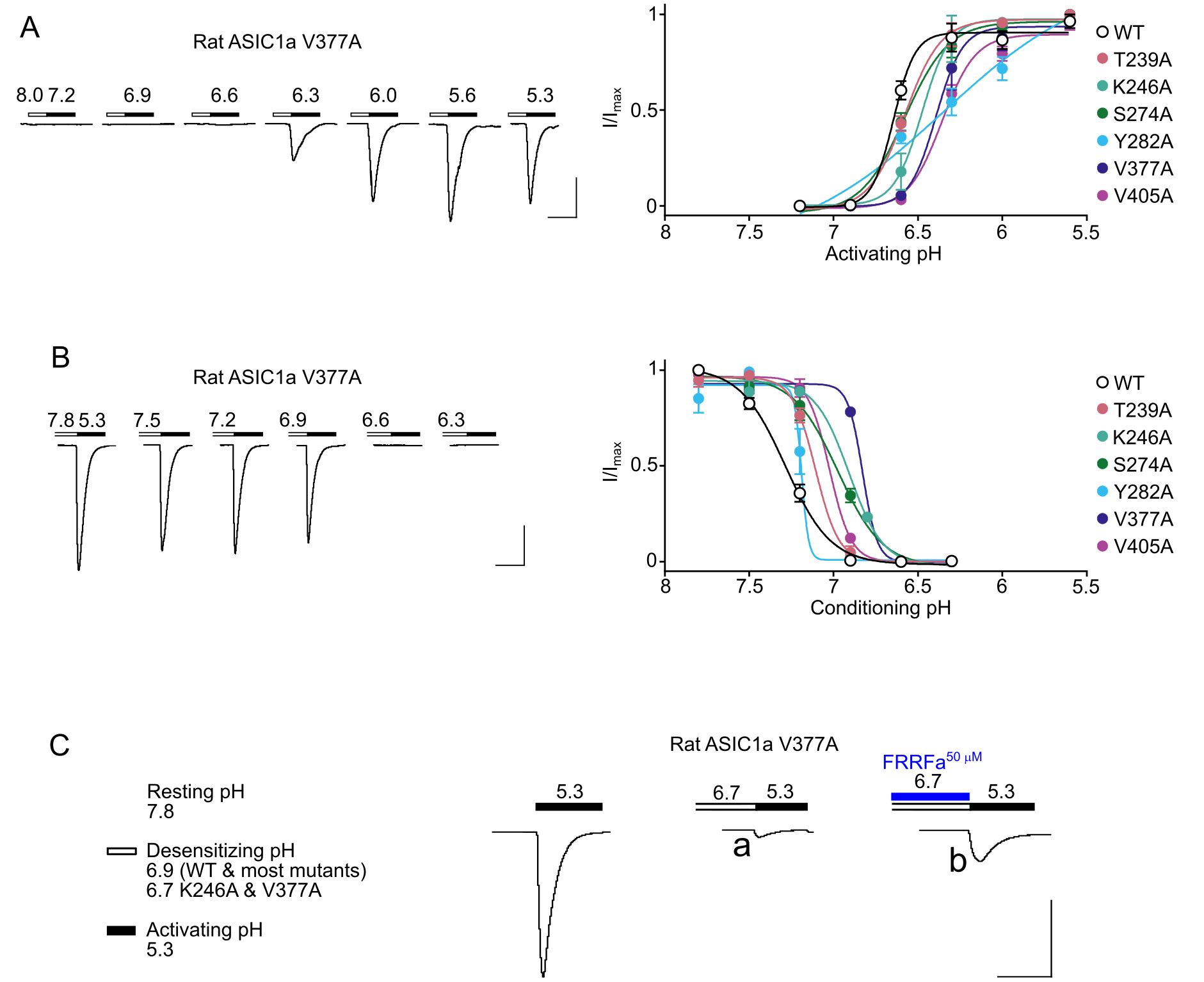


**Figure S4. Measurement of steady state desensitization and peptide modulation of rat ASIC1a mutants.** (**A**) *Left*, example currents at an oocyte expressing a rat ASIC1a mutant in response to decreasing pH. *Right*, mean (± SEM, n = 3-4) normalized currents in response to decreasing pH. (**B**) *Left*, example currents at an oocyte expressing a rat ASIC1a mutant in response to pH 5.3 after pre-incubation in decreasing pH. *Right*, mean (± SEM, n = 3-4) normalized currents in response to decreasing pre-incubation pH. This established that pH 6.9 was strongly desensitizing for WT and most mutants, and pH 6.7 was strongly desensitizing for mutants K246A and V377A. (**C**) Example experiment showing: current response to activating pH (5.3) after pre-incubation in resting pH 7.8; current response ("a") to activating pH after 20 s pre-incubation in desensitizing pH (6.7 for this mutant); and current response ("b") to activating pH after 20 s pre-incubation in desensitizing pH plus 50 μM FRRFa. Dividing current amplitude "b" by that of "a" gives fold-enhancement by FRRFa in Fig. 6B. All scale bars: x, 10 s; y, 10 μA.

**Supporting text**

**Oocyte expression vector and novel cDNA inserts.** For oocyte expression of previously uncharacterized genes, we modified the pSP64poly(A) vector (ProMega) to include 5' and 3' untranslated regions (UTRs) from the *Xenopus laevis* β-globin gene, as described elsewhere (45), and commercially synthesized FaNaC inserts (Genscript) were sub-cloned between SalI and BamHI sites, except for *Lottia* FaNaC_XM_009055314.1, which was subcloned between these sites in the unmodified pSP64poly(A) vector. Sources/databases for these accession codes are described in *Experimental procedures*.

Sp6, HindIII, 5'UTR, SalI, Kozak consensus sequence, XbaI, BamHI, Myc + TAA stop codon, 3'UTR, poly(A), EcoRI

>Modified_pSP64_vector

ATTTAGGTGACACTATAGAATACAAGCTTGCTTGTTCTTTTTGCAGAAGCTCAGAATAAACGCTCAACTTTGGCGTCGACTCTAGAGGATCCGAGCAGAAGCTCATCAGTGAGGAAGATCTCTAAGGTTACCACTAAACCAGCCTCAAGAACACCCGAATGGAGTCTCTAAGCTACATAATACCAACTTACACTTTACAAAATGTTGTCCCCCAAAATGTAGCCATTCGTATCTGCTCCTAATAAAAAGAAAGTTTCTTCACATTCTAAAAAAAAAAAAAAAAAAAAAAAAAAAAAACGAATTCGTAATCATGTCATAGCTGTTTCCTGTGTGAAATTGTTATCCGCTCACAATTCCACACAACATACGAGCCGGAAGCATAAAGTGTAAAGCCTGGGGTGCCTAATGAGTGAGCTAACTCACATTAATTGCGTTGCGCTCACTGCCCGCTTTCCAGTCGGGAAACCTGTCGTGCCAGCTGCATTAATGAATCGGCCAACGCGCGGGGAGAGGCGGTTTGCGTATTGGGCGCTCTTCCGCTTCCTCGCTCACTGACTCGCTGCGCTCGGTCGTTCGGCTGCGGCGAGCGGTATCAGCTCACTCAAAGGCGGTAATACGGTTATCCACAGAATCAGGGGATAACGCAGGAAAGAACATGTGAGCAAAAGGCCAGCAAAAGGCCAGGAACCGTAAAAAGGCCGCGTTGCTGGCGTTTTTCCATAGGCTCCGCCCCCCTGACGAGCATCACAAAAATCGACGCTCAAGTCAGAGGTGGCGAAACCCGACAGGACTATAAAGATACCAGGCGTTTCCCCCTGGAAGCTCCCTCGTGCGCTCTCCTGTTCCGACCCTGCCGCTTACCGGATACCTGTCCGCCTTTCTCCCTTCGGGAAGCGTGGCGCTTTCTCATAGCTCACGCTGTAGGTATCTCAGTTCGGTGTAGGTCGTTCGCTCCAAGCTGGGCTGTGTGCACGAACCCCCCGTTCAGCCCGACCGCTGCGCCTTATCCGGTAACTATCGTCTTGAGTCCAACCCGGTAAGACACGACTTATCGCCACTGGCAGCAGCCACTGGTAACAGGATTAGCAGAGCGAGGTATGTAGGCGGTGCTACAGAGTTCTTGAAGTGGTGGCCTAACTACGGCTACACTAGAAGAACAGTATTTGGTATCTGCGCTCTGCTGAAGCCAGTTACCTTCGGAAAAAGAGTTGGTAGCTCTTGATCCGGCAAACAAACCACCGCTGGTAGCGGTGGTTTTTTTGTTTGCAAGCAGCAGATTACGCGCAGAAAAAAAGGATCTCAAGAAGATCCTTTGATCTTTTCTACGGGGTCTGACGCTCAGTGGAACGAAAACTCACGTTAAGGGATTTTGGTCATGAGATTATCAAAAAGGATCTTCACCTAGATCCTTTTAAATTAAAAATGAAGTTTTAAATCAATCTAAAGTATATATGAGTAAACTTGGTCTGACAGTTACCAATGCTTAATCAGTGAGGCACCTATCTCAGCGATCTGTCTATTTCGTTCATCCATAGTTGCCTGACTCCCCGTCGTGTAGATAACTACGATACGGGAGGGCTTACCATCTGGCCCCAGTGCTGCAATGATACCGCGAGACCCACGCTCACCGGCTCCAGATTTATCAGCAATAAACCAGCCAGCCGGAAGGGCCGAGCGCAGAAGTGGTCCTGCAACTTTATCCGCCTCCATCCAGTCTATTAATTGTTGCCGGGAAGCTAGAGTAAGTAGTTCGCCAGTTAATAGTTTGCGCAACGTTGTTGCCATTGCTACAGGCATCGTGGTGTCACGCTCGTCGTTTGGTATGGCTTCATTCAGCTCCGGTTCCCAACGATCAAGGCGAGTTACATGATCCCCCATGTTGTGCAAAAAAGCGGTTAGCTCCTTCGGTCCTCCGATCGTTGTCAGAAGTAAGTTGGCCGCAGTGTTATCACTCATGGTTATGGCAGCACTGCATAATTCTCTTACTGTCATGCCATCCGTAAGATGCTTTTCTGTGACTGGTGAGTACTCAACCAAGTCATTCTGAGAATAGTGTATGCGGCGACCGAGTTGCTCTTGCCCGGCGTCAATACGGGATAATACCGCGCCACATAGCAGAACTTTAAAAGTGCTCATCATTGGAAAACGTTCTTCGGGGCGAAAACTCTCAAGGATCTTACCGCTGTTGAGATCCAGTTCGATGTAACCCACTCGTGCACCCAACTGATCTTCAGCATCTTTTACTTTCACCAGCGTTTCTGGGTGAGCAAAAACAGGAAGGCAAAATGCCGCAAAAAAGGGAATAAGGGCGACACGGAAATGTTGAATACTCATACTCTTCCTTTTTCAATATTATTGAAGCATTTATCAGGGTTATTGTCTCATGAGCGGATACATATTTGAATGTATTTAGAAAAATAAACAAATAGGGGTTCCGCGCACATTTCCCCGAAAAGTGCCACCTGACGTCTAAGAAACCATTATTATCATGACATTAACCTATAAAAATAGGCGTATCACGAGGCCCTTTCGTCTCGCGCGTTTCGGTGATGACGGTGAAAACCTCTGACACATGCAGCTCCCGGAGACGGTCACAGCTTGTCTGTAAGCGGATGCCGGGAGCAGACAAGCCCGTCAGGGCGCGTCAGCGGGTGTTGGCGGGTGTCGGGGCTGGCTTAACTATGCGGCATCAGAGCAGATTGTACTGAGAGTGCACCATTCGACGCTCTCCCTTATGCGACTCCTGCATTAGGAAGCAGCCCAGTAGTAGGTTGAGGCCGTTGAGCACCGCCGCCGCAAGGAATGGTGCATGCAAGGAGATGGCGCCCAACAGTCCCCCGGCCACGGGGCCTGCCACCATACCCACGCCGAAACAAGCGCTCATGAGCCCGAAGTGGCGAGCCCGATCTTCCCCATCGGTGATGTCGGCGATATAGGCGCCAGCAACCGCACCTGTGGCGCCGGTGATGCCGGCCACGATGCGTCCGGCGTAGAGGATCTGGCTAGCGATGACCCTGCTGATTGGTTCGCTGACCATTTCCGGGTGCGGGACGGCGTTACCAGAAACTCAGAAGGTTCGTCCAACCAAACCGACTCTGACGGCAGTTTACGAGAGAGATGATAGGGTCTGCTTCAGTAAGCCAGATGCTACACAATTAGGCTTGTACATATTGTCGTTAGAACGCGGCTACAATTAATACATAACCTTATGTATCATACACATACG

>Crassostrea_FaNaC_XM_011442205.2

GTCGACACCATGAATGGATATATAGCAAATGGGGGTCAGAATAAAGAACACATGAAGTTTTCACCTGTAGGAAAGTTTAACTATTTTCACGACCATTTGAAGATGCCGCCAAATGACACCCATGCTCGAAAGAAAAATCGAAAATCGGCCATGGCCATAGTGCAGGAACTGGGGTCCGAAAGTAATGCACATGGTCTAGCGAAAATCGCCATGTCACGAAAAACTAAACGAAAAGTGATGTGGTCTCTGCTGGTGATAATTGGGTTTACGGCAGCCGCCATACATTTGTCATTCCTTGTGATAAAGTACCTACAGTACAATGTGGTGGAAGTCTCCGAAATGAAGGACGGAATGCCGGTGGAATTTCCATCGGTAACAATCTGCAACACGCAGGCTTTATCGCTTACCAAACTGAAGGACAGAATGAATGAAACTCCCGATATCAACAACTGGTTTCAATTTATTAGCAATGCAAACTTTGGTGAACACTTCTCGCGAGTGGAGTCTGTTCAGGGATTTTACGAGAATTTATTCCAGGAAGCCAAAAACGTGGGCCACGAAATAAACGATACGTTACTTCACTGCCGATTCAATCAAAGGCCGTGTCACCCTCACAACTTCACTAGATATTTCAACGGCAAGAACTACTATAACTGCTACACGTTTAACAGTAAGATGGGGGACCATAACATGCAGTATATGCATACCACCGGTCCCCAGCATGGCCTCTCAATCGTCATGGCACTGGACAATGACGACCCTCCGCTAGGGGCATACGGACTCTACAACCAAGAGAGTAACATTGAGCACACCGCTGGACTGAGGGTCCAGGTTCATGCCCCCCAGACGATGCCCAGCCCCATGGATCACGGATTTGACATCCCTCCTGGGTATTCGTCCTCCGTGGGTCTAAAAGCCGTGATGAACTCAAGGAAACCTTATCCTTATGGAAACTGTACATATGACAGACTTAAGAGATACCCAAAATACAGGAATACGATATTCTCCTGTTTCCAGTTGTGTAAGCAAGAGATAGTGATTCGCGAGTGTCAGTGTCGGACCTCCGGACTCCCAAGCACTCCGGAACAGAAAAATCTGTCTTACTGCGGGACATTCCATCACTGGCAGAACTTATTCCAACAGCTAAGTAAGTCTGGCTCATTCAATAGAACACACCTGTACAACCAGAAACTGGACTGTGAGGTCAAGGTGCTCCACCGTCTCTCTAACGACCGCAGTTACGAGGTCGGGTGTGGGTGTTTCCAGCCATGTCAAGAAACCTCCTATCAAAAATCCATCTCCCTCTCGTACTGGCCCCTCGAGTTTAACCAGTACAACGCCTTACAGACATTTTATGGAATTTTAGAAGATTCAAATCGGATAAATAATTCCTTCATGTCAGAGGCTTATTACAATCTTCATGGAATTATTAAGAAATATGAGCCACTGGCTCAAAACATGACTGGAGAAAATGCGGAACAATTATTACATGCCGATATTCCGATGGATGAGAGACGAAAGCAGGCCCGTGCCACTCAACTCATTCATCAAAATCTCCTTCGTTTGAATGTTTATTTAGAAGACCTTAGTGTCATAGAATTTAAGCAGATGGCTGACTATGATATAGCAGATTTACTGTCAGATATCGGAGGAACTTTAGGACTGTGGATGGGGATTTCGATTCTGACAATTATGGAGTTAGTGGAACTCGCGATTCGACTGGTTGCCATTTTATTCAAATCGGAAAATAGATATCCTGATCACATGGATGACCACAGTTCAGCCAATGGAATTTTAGAACACTCGACAGAGAGGGCGATATACCCCCAAGATACATATGATCCATACACACAGCCTGAGTTTCAGAGTTTTGAAAAAAATGATTATGGTCGGACTACAAACGATTTACCTCCCGACTCGCCTATCGGATCC

>Octopus_FaNaC_XM_014930938.1

GTCGACACCATGAAGGTAACAGGCTTTGACAAACTGGGTTTCAACAGCAACCAGTTACGCACAATGAGGCAACAGCTACGCAGGCGGAACTATAATGCGCTCAGCATCATCACAGAATTGGCAGCCGAGAGCAATGCCCACGGACTGGCAAAAATTGCAACATCAACACAGACCCCAAGAAAAGTTCTGTGGGCATTAATGGTAATTGTAGGCTTCACTGCCGCCACACTGCAACTCTCTCTTCTCGTCCGGAAATACCTGGAGTTCCAGGTGGTAGAAGTTTCTAAAATGAAAGATGGTATGGATGTGGAATTTCCTGCCATCACTGTTTGCAATATAGCACCAATATCATTGACGAAAACCAAAGAGCTGCTTAGTTCAGACACGTCAGAACTGACACAATGGCTGCGATTTATAGACAAATATAACTTTGGTGCACAGACTGACCGGATGTTCACGGTGCAGAGTTTGTACGAGAACCTCGCAGACGAGGCCCAGCTTTTGGGTAGGGACCTCAATGATTTCCTCATACACTGTCAGTATAATCAGGAAATCTGTAACATCCAAAACTTTACTCGATATTTCGACGGACATTATTTTAACTGTTACACATTCAACAGCGGCCATCAGACCGGCACCAGCCTGTTGACACATGCCACTGGGCCTCAGAGTGGTCTTTCTTTAATTCTTTCGTTAGACAATGATGATCCACCTGTTGGTGGCTATGGCTCATACAATATAAAATCAAACATTGAACACAGTGCTGGAGTAAGAGTGGTTGTTCACCCACCAAGCACGATGCCGAGCCCAGTAGACCATGGCTTTGACGTGCCACCAGGGTACTCTTCATCAGTAGGTTTGAAAACTATTATGCACGCACGTCTTTCAAATCCCTATGGCAACTGCCAAAATAGCCGGCTTCAAAATTCCTCAAAATATATCCACACCGTTTTCTCATGTCTTGAACTGTGTAAACAGCGAATTGTGATGAGCACATGCGGCTGTCGATCTTCAAATTTACCAGAAATGACATCGGCAAACTTCACTTTCTGTGGTTTAGTGGAGAACAAATGGAATTGGAAAGATATGATGAAGAATCCAGAAAACTACAACATCACAAAAATCAACTTGACAGAACTTGCCTGTGAAGAAAGAGTTCTCAAAGAATTTTCCATAAACCGAGCTTACGAAAGCTCATGTAATTGTTTTCAACCATGTCAAGAGACCACATACCAAAAATCAATATCTCTTTCCTATTGGCCTTTAGAGTTCAACCAGTTAAGTGCCCTTGAAACATTTTATGGCGATAAAATCAATGAGACATTTTTACATGATGCTTACAAGATGCTTAAATACTTGTCAAATCTTGGTAGTGTCCCCACACCTGAGTCCCCACCTCCGGAAGAGATCAATATCACTGTGGACGATAACAACAACAGCACCAACTCTAGGATGCATTCTCCGGCTTTACTGTCGCAGTTGCTGGCAAACAGCACTGAGGCACCAAAGCCTGCAGCAACGAGAGACGACTTCATTGAACACGTGAGTGCAAAGCTCAACTCAAGCGACAAAGAGAAACAAATCCGAGCGTCAAACATGATCCGACAGAACCTTCTGCGACTGAACGTTTACCTTGAAGACCTCAGTATCATTGAGTTCCGACAGATGCCTGCCTACGAGCTGGCTGATCTGTTTGCCGACATTGGCGGCACACTGGGTCTGTGGATGGGTATATCGGTGCTGACTATTATGGAGCTTATAGAACTCTTCACCAGACTTCTAATGCTAATTTTCAGCTCGGAAAAGAAAATTCCCAATGCCGACCCAGTCACAAATGGAATGTTGGACCATGACTGTGACTGCCAAAAAAGTAGTATGGAATCACCATTTGGATCC

>Lottia_FaNaC_XM_009055314.1

AAGCTTACCATGGTGCATCGAAACACGGGAAAAAATCAAACAGTACTCTCCCTTGTGGCGGAGCTGGGATCGGAAAGTAACGCTCATGGATTAGCGAGAGTAGTGACATCACGAGAGACCAAACGGAAAGTGATCTGGGCCTTATTAGTGATTATCGGATTTACTGCTGCTACGTTACAATTATCATTACTGGTGAGAAAATATCTTCAGTTTCAAGTCGTAGAGGTGTCAGAGATTAAAGACAGCATGCCTGTCGAGTTTCCCTCGGTGACCGTGTGCAATATTGAACCCATTTCTTTACGGAAATTACGTCTAATGATAAACAATTCTGAAAGTGATTTAGTGCAGTGGTTAACGTTCGTCAACCATTTTAATTTCGGTCCGCAGCAAGCCCACTTAACATCGATCCGCGCTTTTTATGAAAATCTGGGCGATGCAGCAGAAAATTTAAGCCACGATATCAACGATTTTATGATTCATTGTCGGTTTAACCAAGAATTGTGTTCCGTGGCCAATTTCACCAGTTTTTTTGACGGAAATTATTATAGTTGTTACACATTTAATAGTGGCTCTGTGGAAAAATTACTCATGCACGCCACTGGTCCTCAAAATGGCCTCTCGTTAATTCTTTCTTTGGATAACGACGACCCTCCATATGGAACATACGGAATGTATCATATTAATTCTAATATTCTACACAGTGCTGGAGTCCGCGTAGTGATACATGCACCTAACACCATGCCTAGTCCCGTGGATCACGGTTTCGATGTGCCGCCAGGATATTCATCTTCGATTGGATTAAACGCTGTGATGCATACGCGTCTTTCAGAACCGTTTGGAAATTGTACAAATAAGGAGTTAGATGGATACAAGAGTTACAAGAACACATTTTTTTCTTGTCTTCAATTGTGTCAACAACGGATTATTATCAAAAATTGTGGATGTAAATCGTCCAGTTTACCAGAAGTGTTGGACCCAAACATCACATTTTGTGGAGTCGTTCCCAACTGGCAAGAAATTCTAAATCGTTCGACTGAATACAAAAACAAAACTATTGCTGTTCCAAATTTGGCATGCGAAGAAAAATATGTGGTCGAAATGGCCAACGATCGAAGTTACGAATTTTCGTGTAAATGTTACCAGCCATGTGACGAAACGACATATCAAAAAGCCGTTTCTCTCTCATACTGGCCGTTGGAGTTCTACCAACTTCACGCATTACAAGTCTTCTTCGACACAACCATCAAACAAAACTTTATGCACGAGGCATACACTTACTTAGCGCGTCTGGGCAACTTGGACCATTCAATGGACCCCAACGATACCAGACGCAACGAAATTTTACAACAAAGTTACAATCTAAGTGACAAAGACCAAGCAGAGCGAGCGTCAAATTTAATCAGACAAAACCTGCTGAGACTCAATGTATATTTGGAGGGGTTAAGTGTGGTGGAGTTCCGTCAAATGGCGGCATACGGATTAGCTGATTTATTCGCAGACATTGGTGGTACTTTGGGACTGTGGATGGGTATCTCAGTTTTGACAATCATGGAACTGATAGAACTAATAGTCAGACTTTTTTTGCTTCTGTTTAACTCCGAGAAGAAGATCCCAGAACAAGATCCTGTATCCAATGGCATGCTAGAAACTGAACGAGATTACGAGTATGAACGAACTGGTGTGGAATCGCCGGTCGGATCC

>Pinctada_construct_pfu_aug2.0_4392.1_09318.t1

GTCGACACCATGGATTCCGACGTTGTAAATGAGGGACCAACGCCAAAGAAACGGCAACTAGTACGGATGCGAACAAGGGATGTAATTCACAAACTTGGTTCAGAGAGCTATATGCATGGGATTTCGAAGATAATGGCTTCCAATTCATTTAACAGGAGATTGTTTTGGACTATTCTTTTACTATGTGGAGTTATAGCTGCAGTAACACAGCTATCCATCTTGTCTTTAAAATATTTGAAATTCGACACATTTTTTATTACAACAGAAATACAGAACTCGGAGGTAGAATTTCCATCTATCACTGTATGCCCGAATTATGTTCCCTTCAAATATCCATCCGAGGTACTTAAGGACACAGAAATTCAACTTTACGAGGACTGGTTAAGTTTTCTTCTTAAGTTAGAATATCCAAGTACAGAGTACGACCATTTTAGTTCATGGACCGGCTACTATGAAAATCTCCCCGATATTCTACCCCTTTTCGCCACCGAGTGTAGAGATATGATTTTGCAATGTACTTATCGAGGGAAAAGTTGTTCTTGTGAACACTTTGATGAGTATATAAATGCTGAGTATGTTCTTAAATGTTATACTTTCAACGCATCTAAATTTATGACGATACCAAGCATGAAATATGCAACATCTGGACCAGATCTAGGATTGTCGTTGATCCTAGGAGTGGATGATACAAAGTTTGAGGACAAATTTAACATTGTAGGGGATACTTCAAGACCTTATGATCTCGATTACTTGTACGGCTATAGTTCCGGGTTCACAGTTCAGATACACCCTCCCGATACAATCCCAACACCAATGACACATGGTTTCGACATATCAACAAGTCAATCAACAACAGTGGCACTAAAAGAATACATAGAAGGTTATCTACCACATCCATACACTAATTGTTCATTTGATGCATTGCGAAGTGATAAAATATACCGTCGAACATTGTTCACATGTGACCTAATATGTAAGCAAAAGAACGTAATCAAGAACTGTGGATGTACCTCCTCGATTCTGCCTAATATTGGTAAATACCAGATCTGTGGACAATTGAAAAATTGGAATAGCGATCCTAACATAACAGATATCATACAAAATTTGTTGTGCGAGAAAAAGAACATTCTAAATAATACGCCATGTCATTGTTCTCCATGTAAAGAAAATACGTATGATATTACATTATCGGCTTCCGATTGGCCAAAAGACGGTCATGTATTCAAACTACTTGAAGGCCTTCAAGAATATTTAAACGAAAACTTGCGTATGGGATATGATAAATTACGCGATGAAATGTCAGACACTCAAAATTTTGAAAAAATAAATTCTTACATACGGAAAGCAAAGTTAGATTTGGCAAGACTAAATGTTTACTTTAAAGAACTTAATGTTGTGTCAACAGTGCAGATTCCAAGTTATCAATTCGCCAATTTAATGGCCGATATAGGAGGATCACTTGGTCTTTGGATTGGAATTTCTGCTTTGTCCATGATGGAGCTATTAGAACTATTAACATTAATTATGTGTGCATTTAGAAAAAAGAAGAAAAACTACAATGCTTCAAAACAAGACGACGGATCC

>Capitella_Capca1_207658

GTCGACACCATGAAGAAACAGGACAGCAGCCAAGAACTGAGCACGGCTCAGAGGATGAAAGAATCTCTACTGACGTTCTGCCGTCACACTACGGCGCATGGCTTGTGTACTGTGCCGTATTCGTCTAATAAATTCAGTCGCGCTATATGGCTGACACTGTTTTTTGCAGCTTTGGCCGGAATAGCCTATCAGACAACAGGAATAATGACAGCATATTATTCATACCCAGTACAAGAAGTCAACCATATGGACCAGGGTCCAGTTCCCTTTCCAGACGTCACAGTGTGCAACATCGAACCTATAGTGATGAGCAAAGCTCTTGAATTAGCCAATGATCCATCAACAGCCACGCATGAATATAACGAGAAGAAGGTTCCTCTCATACGGGACCTCTTGTTGAGAAATCCGACGTCGATTGCGGGCATGGCCAGCAGAGTTGGTACCTCCAAAGGCTACTTTGAAAACCAACCCCAGAACGAGAGCCGCTACGCCAGTCACTCTCTGAAGGACCTCATCATAGGCTGCTCGTACATACACAAGAACTGTGTGGGAAATCTGGAGGAGAGTTTCACCTACAAAGCGGATGGCACCTATTTCAACTGCTACACCTTCTCTCCAGCCAATAACCCCAACCTATCCGATAACACCGTGGGCCTTGGACCTGAATCTGGCCTCTCTCTTATCCTCTTCATGGATACTGACACAGCAGACCAAACCTTCCCGGGGATAATAGATGTCCAGTCGAATGTGAAGCAAAGCGCGGGTGCCCGGGTGACCGTCCACAATAGAGGAACACTTCCTAACCCCCTCATAGAAGGTTTTGACATAATGCCTGGTCATTCGACGTCAGTCGGAGTGACTGCTACTCGGATACGGCGCCTCTCGGAGCCGTACACTAACTGTACGGACGGAGCTTGGCTGAAATTGAAACCGCACTTTGAGTACACTCCGACTTCTTGCTTGCTGCTCAGCGCCCAAGAATTTATATTCAAGAATTGTGGATGCCTGTCCGCTGAACTTCCTGTACCCGACAATCTGTTTGGTGAGAAATACTGCGGATTTTACGATTATGACAACAACACCTACCTAGAACAAATCGAATGTGAACCCAAGGCTTATGTTAATTTCTACACTGCTACTGATGCCGTCAAGCCAAACACACACTGCCGACCGCTCTGTGATACGTTCCAGTATGATTTCCTTCTGTCGGAAGCATACTGGCCAAAGGAGCGATACGAAGCCAGCTTCTTTGAGGAGTACGTTGCCAGTCGCCCCGATCGAGACTCTCTGCAGGCTTACACATCCCTTGTGAACTACTCAACGACTGAAGACTCAAAGATCAGAAGAAACTTTGCTCGAGTCAACATTTATATGAAGTCCTTAGAAACATTGACTAGATCGCAGATGCCGTCCTATCAAAGCTCCAACTTGCTGTCGGACATCGGTGGAACATTGGGTCTTTGGGCTGGCATCTCTATCATCACCGTTTGTGAGATCATTTCTTTTGCCGTGCGCCTTGCAGTCACTCTGCCGCGAAAGGAAAATATCGGATCC

>Capitella_FaNaC_Capca1_52833_alt_spl_var_Capca1_191096

GTCGACACCATGGACATCAGTATAGAAAAGCAATCGTACACTGCACAAAACCTTTTTACAACAACTGGGCAGCACTGCAAAGTACCTACTGTGCTTGAGGAATTUGCCACTCGGACCACAATGCATGGTGTTCCAAAAGTGATCAAAGCCAAGTCTTCCTTAGCTCGCATCTTCTGGGCCCTGGTCTGTGTATGCGCCGGGGCAATGTTCTGTCTGCAGATGACAGAAGTACTCACCAGGTACTTCTCCTATCCCAAAAAGGTGACAGTGGAAGTGGTCCCCACCCAAGTTCCCTTCCCCTCAATTTCGATCTGCAACATGAGGAATCTGGACGTTTACATCTTAAACACTTTAAATGCGATGTTTATACAGAACGACAACCCAATCAACAACGTCAACAAAAGTGAGATCCTTTTCATCTCGGAGTACATGTCGCTCGTAGCCAAGTACGCTCCGTTGTGGTACCAATACCAGATGGACCTACCGGAGGTATTTCAAGAAGTATTCTCACGAACTACCTTCTCCGCCAACATACGACATGACATCATAGCCTCGGCAGCCGTCCAACTGGAGGGATTTGTAGTGAACTGCCATTACGCGGGACACCGTTGCAACCGGACGCGGGATTTCCTCCGCTTCTTCGATCCGTATTATTTCAACTGCTTCACGTACCAAGCACCAGAAGCAGATGACCTGGATGATTCCCTGAGTGAGGGTATTGAGAACGGTTGGTCCGCGATTCTGCTATCCGGATCAGGAATGCTGGATAAGAACAGTGAGATCCGGATGTTGCCTGGACTTCATGAGTGGCGATCCGCAGTCTCTGCCAGTGAGGGTGTCCGCGTGGTCATTCATCCGCCGCACACGAAACCTTTCCCCTTTGCGGAAGGATATGATGTTCCGCCTGGATTCTCCGCTTCGTTCGGGATTCGCCCGCGACGGAATATACGAATCGGACCACCACATGGGAATTGCTCGAATAACAACCCCTTTGAAATTGGGAATACGCGTTATCGCCTAATGGCATGCCAAAAAATGTGCATGCAGAAGCACATCATCACAGAGTGCGACTGTGCTGATATTGGTTTGCCAATTCTGCCAGATCAGAAGGTTAAACTTTGTCGTAATGATGATGAAATGCCCATTGAGTGTCAGTACAATGCCACCAATAACTGCTTAGACATGTTGATGGCTCTGCATAACAGGATACAATGTGTGAGAACTGTGAAAGCTAGACTCACCAAGAACGCTACGGCGATGGAGCGATGCCAGTGCTTTCCACCTTGTGATGAGGTAGCTTACGACGTCTCCTACAGCCTTTCCAAGTGGCCCGCGTCTGGCTACGAAGGTGATTCCGCCTTCTACGATGTGTTCGGCATTGAGAAATTTGCGGAGAGATTCAACCGGAGTGATACGCAAGGAAAGTTTCAGCTATTCAAGAAGTATTTCACGGTTGACCGTCGTGCGGAAACCATGAAAGACTTTGCCCGACTCAATATTTATATCGCGGACTCCAACGTGGTGAAAACGCAAGAGTCCGCAGACTACACGAGGAACCAGGTCGTTTCAGACATTGGTGGCCAGCTTGGTTTGTGGGTGGGAATATCCATTATAACCCTTGCTGAAGTTCTTGAACTTATGCTGGATATTCTCCGTTATTTTACGTCATCGTCATACATCACGGTATCGACCAGTCCGATGGTCAACAATAACCAAAGCAGAGATATTTCCAATCGTTACAGCATGCCAGAGACTAAATTGGGATCC

>Capitella_Capca1_185559

GTCGACACCATGCCTGGGTCCTCTCGGGGACGCCGCTCCCTCTCCCTCGGGGACGTACGTCACCATGACGACCGAGTCAGCGACGTGCACCGAGTTTTCCAGGAGTTCGCGGACGGCACGTCAATGCACGGCGTGCCCCGCATCATCAACGCCCGTTCGATCCCCGGCCGCTTCTGCTGGTCTGTGATCTGCATGGGCGCCTTTGGGATGTTCCTCTGGCAGTGTGGCATTCTCCTGGAACGCTATTACTCATACCCGAAGAAGGTGATGGTGGAGATTGTCCAGCGACCCGTGCCATTCCCAGCAGTTTCGTTATGTAATACAGACTTCCTGGACCTGGAAGTCGCTCACCATTTGAAGGAAACTCTCATACAAAGCAACGGGACATTTATGCCAACGGAAAAACAAGCCAAGTTCATCGAGAGATACTTCGATTTCTTTCTGTACTCGTTCCATTTACTGTCAGTTTATCAAGAGATAACTCAAGATAAGAATAAGTTACAACAGGACATGGCTGAGGTATATTCCAGGTTGGGATTGGTCGCGAACCTCGGCCACCAGTTGTCCTCAACGGGCAGCATCCAACTGGACAACTTCATCGTCAGCTGCCGTTTCATGGATGGATTATGCAATGTGACGACTGCGTTTGATAAGATATTTGACCCATATTTCTTCAATTGCTTCACTTTCCAACCAAACACAATTCTAAAGTCGAGAGCTTCCAGGCTTCAAGGCGTGGAGTACGGACTGACGGTGCTCCTCTTCACCAAGAGCGCTGGTCAGGTCGGTATGGATAGGGAGGACGCAGAGGAGTTGACTTTTATTCCTGGGCTCCAAGAGTCTGATTCAGCTCTGGCCAGTGGTCAAGGGGTCCGACTGGTCATCCATCCACCTAACACCAGGCCCCACCTAACCGCTGACGGCTACGATATTCCACCAGGATTCTCCGTGACGATTGGCGTGAAAGCGCGCGAAAATGTGCGCATTCGGCATCCTCATGGCAACTGCTCATCCATGAAAAATGTACCACACGTAGAGGAAGAAACGAGCGAGTACACCCAGCCAAATTACCAATACACCCTGATGGACTGCCAGAATGAGTGCATCCAGCGTACCATAATGGAGACCTGCAGCTGCGTGGACAACCGTATCAACATGGTAGAGAACCCGCGCCATCTTCCGTTTTGTTTTAAAGTGCCCAGGGTGTCGGAAAAGTGTAGGAACAATACAATGCGTGCGCTGGTAACGAAGACTCAATCGCAGGACGATGAGAAATGTTTCAAGCCGATCAGGGCGTTCAGTCGGCGCATGGAGTGCCGCAAAGAGGTCTACGAGAACATGACCATCCGCGACCCCGATTCCATGAACAAATGCGAGTGTTACCCGCCCTGCAACGACATTATATACGACTCGTCCTATAGTCTGTCAATGTTGCCCGAGCGCAGTCCAGCGCACTCACAATTTTACGCCAGTGTTGTTCGCTTTGTTGAGGATCTTCCAGAACCTCGTCGAATGCTGTTGGAGAACTCTCTGGGCAAGGACTACAAAGATATTCTCATCAACCGTACAATCCGACTGAATGTGCACATTTCGGACAGCAACATAATCAAAACCACTGAATCCCCTGACTACGAGGCCATTCGCCTGATCTCTGACATAGGCGGTCAGCTAGGATTGTGGATCGGAATTTCAGTCATGACTATCTTCGAAGTGCTGCAGCTGGTTGCTGACATTTTCCGGTTTCTCACCGGAAAAAGCCGGAACGTTGGTGAACTCCGGAATCGAGCCATTGACGATAACTTCCGTAACCATCGCTATGGCAACGGCGACCAAGGATACATACGTCCTTCACATTTGGATCTTGAAAATTGTGAAATCGACAAGCTGACTACAGTGGGATCC

>Capitella_WaNaC_Capca1_212912

GTCGACACCATGGGACTACCTGGATTGCTGGACGCGTTTGCAGGTGGCACCACAATGCATGGTGTCCCCAAAGCCATCAAAGCCCGATCATCTTTTGGACGAGCATTCTGGTCGGTTGTGTGCTTAGCGGCTGCTTCCATTTTCTGCTTTCAGTTTGCTCAGCTTTTGCGGAAGTACTACTCATACCCAAAGAAGGTCAGCATAGAAATCTTACCTTTGGCCGTTCCATTTCCTGCTATCTCTGTGTGCAATATGCGTAATTTGGACATCATAGTGTTGAACAAACTCAATGATATCTTCAAGAGAGAGACAAATAATCTCACTGCACAGAATCTCACCGACAACCAGTTCATCAATGAGTACCTCAAAACTGCGTCCAAGTATCGCCCAATGGTCCTTCTTCCGGATGTTGACCGTGAGATGTTCCAAACAGTACTCACCAGAACTACGTTCGCTACCAATATAAATCGGGATATAATAGCTACCGGTGGAGTTCCTTTCAAAGAGTTCATTGTGACTTGCCGTTTTGGTGGCCATCCGTGTAACCGTACACAAGATTTTAAACACCTGTTTGATCCGTATTACTACAACTGTTATACCTACGAAGCACCTACAACCAGAGTGCCCGAGAGTAGCCTTGCAGAAGGCCTCGAGAATGGATGGTCAACGACGGTGCTAACCGGGAGTGGAATGCTTGATCGAAATGAAAACATTCGTATTATTCCAGGAACTCATGAGTACTTGAGTCCAATGTCCAGCAGCGAAGGAGTTCGAGTGGTCATACACCCTCCAAATACGGAGCCATACCCCCACACTGAAGGATTTGATCTGCCACCAGGATATTCTGTAAGTTTTGGAGTCCGCGCTCGGCAGACAATACGCATTGGGGCTCCGCATGGTAACTGTTCGCCTAGCAATCCTTTTGGAGACCGGGGTAAGCAGTACCGTTTAATCTCTTGCCAGAAGATGTGTTTGCAGCGAGCAATTGTTGATAAGTGTGGCTGTCGGGATATCGCTCTGCCTGGCTACGATCAATACCCAGCGACAGTTTTCTGTAATGACGATTCAGTTTTACCTGATTACTGTCGGGAGAATGCCACGGATGAATGCTTAGACGCGGTGAAAGTAATTTATGCCAGGATTATGTGTGTGAAAGATACGACAGTTGACACAACGCGGAACACAAGTGCAATTCTGGACTGCGGGTGCCACCCACCGTGTAACGAAATCAGTTATGACGTCACTTACAGCCTTTCGAAATGGCCGGCAGAAAGTTTTGATGGTGAAGAGGCTTATATCGATATATTTCACGCCGAGAATTACCCGGCACGTTTCACCGGCCCCGAGGACGCCGAGAAGATTGAGCTTTACGGGAACTATTTCGATCCGTCGAATCGGCGCCGTGCCATGCAGGACTTTGCCCGTTTGAATGTTTACATAGCTGATTCCAATGTCCTCAAAACTGAAGAGTCCGAGGATTACGTTCAATCGCAGTTGCTTTCGGATCTGGGAGGACAGCTTGGATTGTGGGTTGGGATTTCTGTGATTACGATAGTGGAGATTCTGGAGTTAGTCGTTGATTTGTTTCGCTTCCTGTCCACTCGACATGGACCCTACAGTCGAGGAAGTACGTTCTCCAGAGACCCAGAACCCAAGCATCCCGACGATGTGTGCCAAAAATGTGAACACTGTAATGGAATCCGAACCACCTGGTTTGACTCTTTTGTTTCCTTCGGAATGCTTCAGAATGCTTGGGGTGGATCC

>Malacoceros_ON156822

GTCGACACCATGGAGTATACATACAGTAACAGTGATAGGGTGAACAACTCGCCTGGTGGTCCGACACCGAACAACAGGACCCCACAGCACACGAAGATGACTCTATCCAAGGAGAAACTTCACGAACTGGAACAAGAGTTCCTCCACACCAAGACCAAGGACATTGTCAACAAGTTCATGACCACGACCACTGCGCATGGCTGGGGACGTGTGGGCTCGGAGAATAACATCTTCATCAAGCTGTTCTGGGTGGCTATTACACTGACAGCTTTTGGGGCTAATGTGACGCATGTGTTGACTCTGGTGTTGCAGTACAGGGCATTTCCGTCGGAACAAGTGTCTGATGTCGAGCTGTCCTCCATTGAGTTCCCATCTGTTACGATCTGTAACATTCAACCCATGTCTGTCAGTACTGGCCTGGAGATGATGCAGGACCCATCCACACAGCTCTATCAATGGGACAACCTTACACAATTCTACTATGACATGGCCAAGAAGGACGGTAACTTATCCTACGAAGAGGAGTTGAAATACAATCGTCTCAAGCAGCCAATTGGTTACTTTGAAAACATTGGTGATGAGGCCACAAGGGTAGGTCATCAGCCCCGAGATTTCCTCCTTAGTTGTACGTTTGGATATAGGCAATGCACTTGGGATAACTTCACATTCTTCCAAAGCCCCACATATTATAACTGCTACACGTTTAACGGTAACAAAAGTAGTGAGAATCTAGTGGCAAGAACAACTGGACCACAAGAAGGACTGAGTCTGATCCTTTATCTTGAAAGTGACAATGGTGATGAACTATACAATGGCACATATCATACCTTGTCAAATGTCGGCAATGCAGCAGGTGCTCGGGTGATAGTTCATCCACCTAATTCCAGACCCAGTCCAGTTGACCAAGGTTTTGATGTACCTCCTGGCTTTTCTAGCTCTGTCGGTGTCAAAGCTACCCGTTACGAGCGACTCGGTGAACCATATGGCCCATGCGTTGAAGACACCATGCATGGTAGTGATCTCTTTGTGTATGCCACAGACACATGTGTTACTTTATGTCAGCAGCGCTATGTGATGAATGCATGCGACTGCGTCAGTTCCCTTCTACCCATTCCAGAATATAACGAATCAAATTTAAAATACTGTGGGAACTTTGACGCAGCACACCAGGAATATTTCTTCTCAAATCTGTCATGTGAAGCGGCATCATTAGCTGAATTTGTCGCAGAAGATAACGTGCGAGAAAGTTGTGGCTGTCATCCACCATGCGAGGAATATTCATACAAAACTGACGTTTCATATTCATATTGGCCACTTGACTTCACCCAACTCAGTTTCTATCAGAGCTATGTATTAAATCATCCAGACGTGGACAATCTCAAAGCGTATCAAAATCTTGAGAACTACAATACATCAGAGTTGATCTCGACTGGTCTCATACGAAAAAACTTCTTGAGACTTAACGTGTACCTGAAGGATCTGATCATTCAGGAATACATTCAGAAGAAGTCGTATGAGATCCAGAATCTGATCAGCGACATGGGTGGGACGTTTGGCCTTTGGATCGGCATGTCTGTGATAACATGGTGCGAAGTGGTTGAGCTGCTGATCCGGTTAACATCAAGGAGTGTTCGAAAACTTGTCCACAGCGGAGAGATGCAGGGTTCGCCTAAGCATTCACAGCGTGGCTCAGGGGTGAACTCTCCCAATAGTATGGACAACAAGGTGTCGCCACGGATGGACCCTCATACTGCCCATGCGCTCGGACTGCAGGATTGTATTGTGTATAAAGAGGTGCAAGTGGGATCC

>Malacoceros_FaNaC_ON156826

GTCGACACCATGCCTATCAAGGACGTTTTGGAGAGTTTTGCCAACCACACGAGCATGCATGGCCTGCCTAAGGTCATCAACGCACGGTCAACACTCACAAGATGCTTTTGGGCGGTGATTTGCGTTGCTGCGGGGGCAATGTTTTGTATGCAAATGACTGAAGTGCTCACGCGCTACTTCTCATACCCCAAAAAGGTGACAGTAGAAGTGGTGCCCACCCCAGTGCCCTTTCCCGCAATATCAATGTGCAACATGAGAAACCTCGACTTTGACGTTCTCAACACAATCAATCGTAAATTCATCGAGGATCACAGGCCACTGAACCACATCAACACATCAACCAACCCTTTCATCCGCGAGTACATGAAAACAGTCGCCAAATACGGTCCACTGTGGTACGAGTACCAAGAGGAGTACCCGCTGATCTTCCAGGAGGTGTTCTCGAGGACTACATTCTCATCCAACATCCCCGAGGACATCATATCATTAGCAGCAGTCCAGCTTGATGAGTTTGTCGTCAATTGTCATTTTGGCGGCCATGGTTGTAATCGTACGAGAGACTTTGTTAAATTTTTCGATCAGTATTATTTCAATTGTTTCACCTACACAGCCTATCAGGATAAAAATGAAGATGAGTTTTCATTATCCGAAGGTATCGAAAACGGTTGGTCGGCAATACTATTTTCAGGAAGCAATATGTTGGATAAAAATGAGGATATTAGAGTGTTACCTGGGTTACATGAGTCGCAAAGCCCCGTAAGTGCTAGTGAGGGAGTGAGGGTTGTTATACATCCTCCAGATACCCAACCATTTCCATTTACAGAGGGTTATGATGTTCCCCCTGGCTTCTCCGCATCGTTTGGAATAAAACCTCGCATGAATATACGCATCGGGCCTCCACATGGCAATTGCACTAACAAGAATCCATTCGGCAACAAAAACAAATCTCGTTATCGCGGCATCTCGTGCCAGAAAATGTGCTTGCAAAGCCATGTGATCAAAACCTGCGGTTGCTCCGACTCTTCCCTCCCAAAGGTGCCGGATACCGACGTGAGGCCGTGTAGGAATAATGATGATTTCCCTGATTCATGTATGTTTAATGCCACAAGTGAATGTCTTAGAATACTATTTCGATTGTATGCACGGATACAGTGCGCTCGGCAGACGCGTGACTGGATAACTAAAAACACAACATTACTTGTTGATTGCCAATGTTTCCCCCCTTGTGATGAAGTGCTTTATGATGTATCTTACAGCTTGTCTAAGTGGCCCGCATCCGGATATGAGGGGGATGCTGCCTATTACGATGTATTTTATATTGAGGGTTTTAAGGAGCGGTTCAAGACTACGCCGAAATACGAAAAGATCAAGACGTACTTTAACGACTCGACGCGAGAGACTACAATGAAGGATTTTGCGCGACTCAACGTCTACGTAGCAGACAGTAATGTGATAAAGACACAGGAATCTGGAGATTACACAACAAATCAGCTAGTGTCAGATATCGGAGGCCAGCTTGGATTATGGGTAGGTATCTCCGTGATTACGCTGACTGAGGTGATAGAACTGTTATGCGAGATTATGCGCTATCTATTGTCAGCCAAAATGACGACCCCGAAAAGAGACATCGAGCCGGCCGGGGATCGCATGAGAAATGGGAAATATAGACCGGCTGCCATGGAGGGACTTCCCCCTACACAATACGACGGCTACGATAGCGCGGATCTTATGTTGGCGTACTATCCGGACCCGCTGCGTAGGGGATCC

>Malacoceros_ON156827

GTCGACACCATGACATTAAACGACAGTTTTCTCAGTGCGAAGGTGGCATGTTCTAAGTCCAAAATGCCAGCCCCTAAGGATGTTCACCGTGTGTTCCATGAATTTGCCGACATCACATCTATGCATGGCGTGCCACGCATCATCAACGCTCGTTCCGTGCCTGCTCGTATCTTCTGGTCTGTTATATGCTTAGCCGCATTCGGAATGTTCATGTGGCAAAGCGCCATCCTCTTGCAGCGCTATTATTCCTATCCAAAGAAAGTTAACATGGAGATCATCCAACGTCCAGTCCAATTCCCTGCCGTCTCAGTGTGCAACTCTGACCCGCTAGATTTGCTTGTGGCTGATAGGCTAAGTGCATATCTTGATGAAAGGAATACCACATACAATATAACAGACGAGGAAGAAGATATACTGGTGGCAATTGAAAACAAGTATGGATCATTCTTTACAGCAACAATGGGCTTCTTCCAAGTTTACTCACAGAACATGCAAGCAAGAGGAGGAGAAATGAAAGCTGATAGTGTGATTTTAGAAATAAGCTCTCGCTTAGGGCTAACTGCAAATCTAGGGAGGAATTTATCATCACTAGCAGGAATACAATTAAGAGATTTTATTGTAAATTGTAGATTTATGGATGATGAGTGTAATATAACAACTGCATTTATGAGAATATTTGATCCCTATTTCTTTAATTGCTATACCTTTAGACCGGAGACTATACTTCCGAGTCGTGCCACCCGTTTGCAAGGTGTGGAGTATGGTCTTTCATTGTTGCTATTCACAGGAAGCGCAGGTCAGCTTAAGGTTGGAAAAGAGTTGGAGGGTCTGATCATTCCAGGTATGCAAGAAACAGATATCGCATTAGCCTCTGGGCAAGGGGCAAGAGTTGTAGTGCATTCCCCCCACACTCTCCCTCACCCTGCGGCAGCTGGCTTTGACGTGCCGCCCGGGTATAGCATAGAAATAGGTGTTAAGGCCAGAGAAAACGTGCGCATTCGACATCCGCACGGAAACTGCTCTGATCAAGCAGCTAACAAGTCCAACTTCCGTTACACACTCATTCAGTGCCAGCAGGAGTGCATGCAAAATAGGATAATGCAATCTTGCAACTGTGTGGACAATAGGATACCCGAGCCAGAGGATACATTAGGCTTACGCTATTGCTTCCAATTGCCGGAGTTACCTGAAACGTGTCTGTTCCAACCGTCCTCTCAGCTCTGCTTAAATATCATCCGAGATTGGACTGACAAGATAGATTGTCGGAAAGAAGTCTACGAAAACATGACAATAAGGGACCCGGAGGCTATGGATAGTTGTGAGTGCTACCCGCCGTGCAACGATATTGTATATGACACGCTTTATAGCCTGTCAACGCTTCCCGAGAACACAGGCGAGCATTCAACCTTTTATTCCGAGATAGAAAAGTTCCAAAAACTCGGGTTATCCAGTGCTAAAATCGATCACTTGAAGTCACGGTATGGCACTGACTTTGAGACTAAAATGGCAAGTCATATCAGCCGCTTAAATGTTCATATAGCTGATAGTAATATCATCAAAACTACCGAGGCACCTGATTATGAAGCAATTAGACTTGTAAGTGACATTGGTGGACAACTTGGGCTCTGGATTGGTATATCTGTGATGACACTTTTTGAGGTTTTGCAGTTGTTTGCGGATGTGTTTCGGCAGCTAACAGCCACAGGTCGTCACATAGCCGAGAGAGTACCAACACGCGAGCCCATCGTGGCCACCAGCCTCAACAACAGCTACGACCCTCACGAGCCCTATGCAACAGACCCTCGGTTACGCGCAAACATAGAACAAGGCATCCCGACCACGAGACGCTTTCACAATTACAACAACGATCGGATAGTCGGTGGATACGATCTGGATATAAATCAACGCTACGAGGTGGACAAGCTCACCACTGTGGGATCC

>Malacoceros_FaNaC_ON156825

GTCGACACCATGGCGATTCGTGATGTAATGACAAAATTTGCTGAACAGACTACTATGCATGGCGTGCCAAAGGTCATTAATGCCAAATCAAGCATGGGTCGTTTGTTCTGGAGTTTAGTCTGTCTGGCAGCCGGAGCTATGTTTTGTCTACAAATGTCTGAAGTTTTGCAGAGATATTTCAGTTACCCAAAGAAAGTGACTGTTGAAGTCGTCCCCACTCCAGTGCCGTTTCCAAGTATCAGCATCTGCAACATGCGAAACCTAGATGTTCACATTCTCAACACTCTCAACCGAATGTTCATAGAAGACGACAGACCATTCAGCAATATCAATAAATCGGAACACGAATTTATCCGAGCTTACATGAAAAAGGTGGCTAAATATGCCCCACTCTTTTGGAATTACCAAGATGAGTACCCAGAAGTCTTTCAAGAGATATTTAGTCGAACTACTTTCAGTGCAAACATTGATCCGGAGGTAATCGCATTGGCCGCTGTTCAGCTTGAGGGCTTTGTTGTCAATTGCCATTACGCTGGACATCGGTGCAATAAGACTCGAGATTTCTATCGGTTCTTTGATCCATACTATTTCAATTGCTTCACTTATAAAGCTCACGAACCGACAGACATCGAGGATAATTTATCAGAAGGGATAGAAAATGGGTGGTCATCTATTTTACTAAGTGGGAGTGGCATGTTGGATAAGAACGATGAGATTAGAATGTTGCCAGGATTACATGAGTGGCGAAGTGCTGTATCTGCCAGTGAAGGAGTAAGGGTTGTTATACATCCACCGAGTACAACCCCGTACCCATTCACAGAGGGATATGATGTTCCACCCGGGTTTTCAGCTTCATTTGGCATTCATCCTAGACGTAATATCAGAATAGGACCTCCACATGGAAACTGTTCTGATAAGAATCCATTCGGTGATGGAACTGAGCGGTACAGACTGATGGCATGTCAGAAGATGTGCATGCAACATTATATTGTAGAAACATGCGGTTGTGCGGATGTTGGCCTCCCGAAACTACCCTTACAAGCTAATATCTCATGGTGTAGAGACGATGATAACTTTCCTGATGAGTGTATGTTTACCGCTTCAGAGGAGTGCTTACAATTGCTAATGCAACTGCACAACAGGATTAAATGTGCTCGATCAATAAAATCTAAAATTACTAAAAACACAACTGCCATGGAAGCATGTAATTGTTTCCCACCTTGTGATGAGGTCAGCTATGACGTGAGTTATTCACTTTCAAAATGGCCGTCAGCAGGATATGAAGGGGATGCTGCATACTTTGATGTATTTGGAATTGAAAAGTTCAATGAGAGGTTCAATAAAACTGGCACACAGGGCAAATATGAACTATTTACAAAGTATTTCAATGTCTCAAATCGTGAGGAGTCAATGAAAGATTTCGCTCGCTTGAATGTTTACATAGCTGACAGTAATGTTGTGAAAACTCAAGAATCTGAGGATTACACAAGAAATCAATTGGTGAGTGATATTGGGGGTCAGCTAGGTCTCTGGGTCGGCATATCTCTTATCACCCTAGCCGAGGTGCTTGAACTAATTATTGATCTGTTTCGTTTATTCTCCAAACATACCTACCGAAGCGTTCCAGTCATTCGACAAAGTATAAAATATAAAGACAAGCGGAACGGTGCAGAAATGAACTATGACACAAGATACAGTCAGTCAAATGGCGGACCTCATGCTCGTTATTTACATCATGGACACTCAATACCAAAGCATCCGCCAGAGTTACCTGATACAAGTTTGGATCC

>Malacoceros_WaNaC_ON156824

GTCGACACCATGCCGCTCGGTCATATGATGCAGATGTTTGCCGGGGGCACCACAATGCATGGTGTCCCCAAGGCAATTCGCTCAAAGTCGTTAACCGGACGGATTTTCTGGAGTTTTATCTGTTTAGCAGCCGCCTCGATGTTTTGCTTGCAGTTTGCGCAACTTTTGCAGAAATATTACTCGTACCCTAAGAGAGTAACTATTGATATTGTCCCTTCCCCTGTGCCATTTCCAGCTATTTCGTTATGCAACATGCGTAACTTGGATATCATCGTCCTCAACAACCTTAATAAGATCTTTAAAGAAGCAGCTGATCCCTTAAATTGGGGGAATTACACATCTGATCCGTTTATCAATGCGTACATGAGCGTGGTTGCTAAATATTATCCCATGTTTGTCCGGCCGGATATAAACATGCATGTCTTTCAAACCGTTCTCACACGCACATTGATTGCGACTAATATAGAGCGCGATATGTTAGAGCGGGCAGGGGTGCCATTTAAAGAATTTGTAGTGACGTGTCGCTTTGGGGGCCATGACTGTAATAGGACCAAAGATTTCCGTCAGTTCTTCGATTCATATTATTACAATTGCTTCACATATATTTCGCCATATACCCCTCAGGATCATGATAGTATTTTAGCTGAGGGGCTCGAGAACGGATGGTCAACGGTTGTTTTGACTGGAGCAGGAATGTTGGATAAAAATGAGGAGATTCGAATGATCCCAGGTACGCATGATCGAGTGAGTCCGATGTCAAGTAATGAAGGAGTGCGGGTTGTCATACATCCCCCAGACACTGAGCCCTATCCACATACAGAGGGATTTGACGTTGCCCCCGGTTACTCTGTTAGCTTTGGGGTTAAAGCGCGTGAGAACATCCGCATCGGCCCGCCGCATGGCAATTGTTCCAATAGCAATCCATTCGGCAACGAGTCTTCCGCCTACAGACTCATATCTTGTCAGAAAAAATGCTTGCAAAAATATATTGTATCTCAGTGCGGGTGTAAAGAGATCTCAATACCCGGGCACGATGCCATGTTCCCTGATTTAGACTACTGTACATCGGATAAAGACATCCCTGATCACTGCCGGGAATCGTCTGATGATGATTGTGTTAATGCACTCTTCAAAGTGTATCACCGCTTCCTCTGTGTGCGTAACGCATCGAAGCACATGACGACAAATGCATCCGCGACACGAGAATGCGGCTGCTACCCACCTTGCCATGAATCAAACTATGACATAACTTACAGCTTATCTAAATGGCCGGCTGAAAGTTTTGATGGAGAGGAGGCTTATATCGATATATTCGAAACTGAGAACTATCCCGGTAGGTTTATGGAGCCGGAAGATGCACAGAAATTCGATCTTTATGCCAACTATTTCGATGCCTCAAACAGACGGACTGCGATGAAGGATTTCGCTCGTTTAAATGTGTATATAGCGGACTCGAATGTGCTAAAAACTGAGGAGTCTGAAGAGTATACACAGTCTCAGTTATTGAGTGATATAGGGGGACAGTTAGGGTTATGGGTGGGGATTTCTGTAATTACACTTGCTGAAGTATTAGAATTAATTGTGGATGTTGTAAAGTGGGTGGTGCTGTCACATGGACCATGTTCACAGGGTCGGACATTCTCCAAAGACCCTGAAATCGGTGAACAAGAACGGGGACTAAATGGGGAAAGTAAATCAAAATCAAGGAACTCAAAGTCCTCAAGAAACTCAAAACGTTCAAACGGGAAAAACAAGAAGCGGCAAATGAACGGCACTATTCCGCTCACGGCTACCACCGTGTATGAAGAAGAGAATGAGTACCAGGAACCGGTCATGGATCC

>Malacoceros_ON156823

GTCGACACCATGGTTGTGTTGTCCATGATTCGCGATTATGCCAGCAACTCAACAGCTCACGGCCTCCCTAAACTTGCCAAAGCGAGAAGCGCGCGTGGGAAGGTGTTCTGGACATTGATATGTTTTAGTGCTCTGTGCTTTCTTCTCATACTTGGTACATCACTTCTTAGGAAATACTATGCCTACCCTAAGTCAGTTCGTCTGGAGTTGGCAAATCAGCCTATTCCCTTTCCGGCGGTCTCCCTCTGTAATCTGCGACCAGTAGATGTCTATACCATATGGAAAGCTATCTTTAATGAATCAGGTGAGAGACAATGGGACATCGGAGAAGACTTTGATTACGACGGACGAGATCCACCCGATGCAGATACCTCCATCTTTGAATCACATCTCATACGCTTTGCAAACAACTTCAAGTCCTACCAATCCTACTCAGACATCAAAAGTGAGAAGGACCCTATCAAGCTATGGAAGATGGAACGAGAGTTATTGAGTCGTATGACAATGATTGGGAATCTAAATCTCACAGCTGCACAACAAGGAGGGGTGCAAAAGGAACAGTTCATTGCCAAATGTGAATATGGGGGTGAACATTGCACAGCCAAAAATTTCTCGCTGTTCCAAGATCCGGCCTATCTGAACTGCTATACATTTAATCTATCAAAGAACCTGGATTCTATTTATGAGGGTCCTGATAATGGATTGTCTGTTGTTCTCTTTGTCCCTGGCCCATCAATTATGGGACAAGATATGGATAAAGCGGCTGATATTGCCCTCCATCAGGAATTGAGTCTTGGCGGGGAGGGAGTTCGTGTTGTAATACATGAACAAAACACAGTCCCTTATCCACTCACTGAGGGGCTTGACATCCCCCGGGGTGTATCAGCCAGCGTAGCTATTAAATTGATAGAAAACGATCGACTCGGCCCGCCTCATGGTAACTGCACTGACAAAAAGACGATTGATGGTTTCATAAAATACAGTTACACAATGGCGTCTTGTAAGAAAACATGTCTTCAGAAACTTGTGATGGAGACATGCAATTGTGGTGATGTCTCCCTACCCATTTGGAACACCAACCTTACCTTATGTACGAAATTTGATGAGTTACCTGCTGAATGCCAAGGGAGGGAAAATATAACAAGGAATATTGCGCATTGTGAGGTCTTATTTGATGAATGGTTCCAACGGGTAGGTTGCACTAAATCTACTAAGGCAAATATATCCAAGAATTTATCTGCTTGGACTGAGTGTGATTGTATGCCTCGTTGTCATGACACTGAGTATAGTTTATTCTACAGCCTCTCTGATTGGCCGATGCAAGAACAACAAAGAGATGTTGTCCAGGAACTACTTTATGTCGATCGCTTCATCCACAACTTTCCACCAGAAAAACAAAAGCAGTACTTTGGCTCCATTGATGTAAGAAATAGTTCCCCAACTTATGATGACTACCGCACATTTGTCGAACAGAAAAACTTGATCCGATTGAACATCTACATTTCTGACACCAGTGTTGTGAAAATAATGGAGACGGAAGCCTATAGTCTGACTCATTTGGCGAGTGATATTGGGGGACAGTTATCTCTCTGGATCGGGGTGTCTATTATCACTATGGTAGAGATAATTGAGCTTATTTGGGGGATGATCAAAATCTGTGCAGCGGGGACAAATAAAACAAAGGAAGAGGAGAAAGTGAATGGAACAGACATGGAAAAACAGAAGATGAACTATGGGGATGAAGTTGGATCC

>Malacoceros_ON156821

GTCGACACCATGGATGACACACGTATACAAAGAGGGGAAGTTAAAGGAACACTTTCTTCCTTTGCTGAAAGTACAACTGTTCATGGTCCGTGCCATATCGCACAAGAGAGCAATAAAATTGGCAAAGCATTCTGGGTAATAGTATTTCTTGCTGCACTAACTGGTGTCACTATACACCTTTACCTGACTCTGACCGTGTATTACAGCCATCCAACTCATCAATCTCTTTATATCGCTCAAGAAGCTCCGCAGTTTCCCCACGTGACATTCTGTAATGAAAATCCCATCTCTCGAGAAAACATGGATGAATATGTTAGTATTAATCCAAACTCGAATACAACATGGTTTAGAGGACAGATTGCACAACTCTCATCAAAATTCCAAGAAGTTGTGGAGCAAATTCCACACATGGATGATTTATTTGCCTCAGCTGCAAGCTACTTTGAAAATATAGGGCGCCATGAAGCCACAAATATTGGACACAAATTTAAGGATTTTGTGATTGATTGCACATATGCAGGAATACCATGCAGCAAAGAGGATTTTGACTTCTTTTTACATCCAATATATTTTAACTGCTATACATTCACAGGGAAACAAGTTAAAAAGAAAGCACAAGAAACTGGTGGAGGTCCTTTTCTTGGTCTCTCAGTTGTACTGTTTCTTGAGAAGAACCCATCACAGATTCAACTGTACAATAGATATTCTCCAGTTGGCAATGTTGTTGGAACAGTTGTTCAGTTACATTCTGCAAATGACTTTCCCACCCCTCACATAGAAGGTGTCCATGTTCCTCCTGGTCATTCCTCTGCCATAGCCATGCACACAGAACAACTCAACAAACTTCAACCTCCATTTGGCAATTGCTCAGCTAGACTCCTGCATACACCAGCAGATTACCACGTCAGCTACACATCCTCTCTATGTCTGGCACTTTGCCAGCAGAATCTCATCTACCAAGACTGTGGCTGTTACATGGCTACTCAACCTTTCTATGGTCTTGAAAACCCAACATGTGGTACTTTCCATCTGAATGCGAGTGACAGTGAGAAACTTAGTGTGTATGCCAAGAACATGAAGTGTCAGAATGAAGCCAGCAGGAATGCCAGACTTCGTAGTAGAGATAGGAATATTTGTGACTGCCCACCAGAGTGTAATAAGACTATTTACTATAAAAGTTTGTCACAAACAGTGTGGCCAGCTGATAATTATTACCGAAATTTCTTGGCTGAATTTCTTGATGAACGACAAGATAAGGCTGACCTTATAGCATACAGGAACTTACATCACTTGATAGATACAACATCTGATGAGGTTGAGCTTACCAAATCCATAAAAGAGCAATTTGCTCGAGTAAATGTCTACTTCTCTGACCTTGAAGTAGTTGTAAGACAGGATGTGAGGTCATATACACTTCCACAGCTGTGGTGTGATATTGGAGGCACTATGGGTTTATGGGCAGGTATGTCTGTCATCACTTGTCTTGAGGTTCTGCAACTTCTTGCTAAATTACTGTCTACATTGTGTAAATCTAAGAGCAATGATAAAACCGGGATTACTTCCATCAGAAATGCTCATACTGAACTGCCTGTCAAGTCGGATCC

>Macrostomum_Mlig049925.g2

GTCGACACCATGCAAGACCGACTCCGCACGGTGACCGGTGCCGCCCAGGAGGTGGCTGGCATCACGAGAACCCACGGCTTGCTGCACATCTTCCTGTCCAGAGGCACAGCTCGACGGCTCTTCTGGCTGCTTCTGTTCGTAGCTGCGCTGATCGGCTGCACCGTCCACCTGGCAAAGTTGGTGCAGAAGTTCACCGAGCAGTCCGTTGAGTCGCAGCTGAAGCTGGGGAGCGAACGAGCCCAGTTTCCCGACGTCACAATCTGCAACTTCAAGCCCGCTTCTGCTAGCTTAATAAGGTGGAATATTTATCACGACTACCTGAATAAATCCTGGGGCAACTTCGACAACTGGTTCCAAGAGGACACCAAGAAGCGCCCGCAAGAATCGGATGAAAGCTACGAAGACCGACTTGAGACGGTGTGCAAGAAGCTCCTCGACGTGATGTGGGTGTCAGGCGACACCAGAGACATCTCCCACCTAGATAAGACCATGCTGCTGAGCTGCCGCTACAACAGCGAGCCGTGCAGTCACAGGAACTTCTCGCTTGTCCAGACCAGCCGGTTCTGGAGCTGCTATACGTTCCACCCGGACCCAGGGCAGTCGTCCAGCGGTTCTGGGGGCAGGGGAGCCGAGCTGGACATGGTGCTCTTCACCGATGGTTATGCCGAACCGTCTATCCATCATTATGAGTTCCTAAATTCCGAATCTGTGGGAGTCTGCGTCAATCGAATAATTTTGGGTCAGGATGCGACGAACAGGTTGCTGAGAGGCAGTGATCCTCAGTCAGAAGGACTGCGACTTTTTATTCACGAGGCGCACAGCTACCCGATGAGCCAGACGGAGTTCGTGGACGTGGCCTCAGCCACCAGCACCTCCATCAAACTCAGGCCGATCCACAACCGACTCAAATCGACGCCGAGCCGCAGATGCTCCGAGCCTCCAATCGAAACCATCAACTATGTCAGGCACTTCTCCAACGCCTCGCTGAATGTGGTCACCAAGGCCTACTCAAAGTCCGTCTCCGATTTCGTAGTCGAGGCACAGCAGAGCATCCTGCACAGCAACTGCGGCTGCTATTCGCACCTGCTCCCGTTCTCGGTGAATACGAGCGACCTGTGCTACTTCGCTCCACCGCAGGAGTGGATCACCCCATCGGATGCTGTGCTCAAGAGAATTGACTGCCATGATCACTGGTTTGAGCACGCACAGAGGCAAAGCGATGAGCTGGCGAAGGAGTTCCAGCACCGGATGTGGTGCCAATTCACTCAGCAACGGCCTTGGCAGCAGAGCCGCAGGTGGCCCCCGTTCAGCGCCATTCAAGAGATCTGGGAGAACCTGATGGTGCCGCAAGTCAGACATGGAGTCGAGTTCCTGCCCGAGGATGGCAAAGAGCATCACATATTCAGGAATATGAATCAATCTCGACAATACAGCCAAATTTTACCATTTCTAAACGACGATGACTGCATGAGTTTCTTTTGCAATCACAGCCATAAGGATTACTATTATTGCGATACTTTACTGATTCAGCTGTATTTGAAGCAAGCGTCGGGTTGCATCGAGCGGTACACTTTGTCTCGCAGCCTGGCGTCAGTCAGCGTCAGCTTGCAGAGCCCGGCAGCTGACGTTTACAAAGAGAAGGCCGGCTACCACTGGACTGAAGCTCTGTCCGAGGTCGGAGGAACTCTGGGGCTCTGGCTGGGAATCAGCGTTGTCAGCACCTTCGAGCTGCTCGAGTTTTTCTACATTCTGGCGCAGCGATGTCGCGGCGGAACCACCGGCTCGGAGTCCGAGGGGTCTCCAGACGGATCC

>Macrostomum_Mlig051885.g1

GTCGACACCATGTACAGTCAGGAATCGAACCGACCAGTGGCGAGCTGGGCTGCCCTGCGCGGCGTGTTCATGCACCTGAACAGGTTCGCTCAGCACACCAGCATGCATGGCGTTGCGCACCTATTTAGAGCCGACAGCTACGTTAGGCGAGCATTCTGGTTGCTGCTCACCTGTCTCACCAGCATGGCCGGATGCTATCACGTCTACCTGACTGTGCACCAGTTCAGCCTGACTCCAGTTAGCACGCTCATTCTTAATGAAGGAGTAGACACCATATTCCCGGACGTGACCATCTGCAATCTGCGCCCGATGCCGCAGTACCTGCTGAACCATGATAGTCCAGCTAGACGGCAGTTGGAGTCCAACATGCTTGACCTGATGGTGGCTCTTAGGCGAGTCTACGCCAAAGGTGGCGACATATTCGGCACGCAGAAGTACTTTCACTGGTACAATCTGTTGCAGCACTACTGGGCGTCAGTGGACACGGCCCATCTGGGCCACAACCTTAGCTTGTCGATGGTGGCCTGCTTCTACAAGAAGGCCCCGTGCAGACCAGCGGATTTCAAGCTGGTGCAGAACTCGCACTATTGGAATTGCTGGTCATTCCGACCCAAGGATCGAAGCGTGCAAGGCACCGGGCCCAATGACGGCCTGAATATAATCCTGTACACCAGCACAATGCCGCTGGATGACTCCAGCCAGCCGGATCACGTGGTAGAATATCCGCCGCCAAAGCATCGTTCGACGTTAAGACAATTGGACACGGTGTTCGGCAAGGCCGGCATGCAAGTGTCCAGCGGCGTCAGGCTGCTCGTGCACGAACCTGGCACGTATCCGCACGTGTACTGGGAGGGCGCCGACGTTGGCAACGGTTGGAGCGCCGACTTGCGCTTCAAGATGAAGCGAAACGTCTACGTAAACAGAACGGGACATAACTGTGTTGAAAACTACGGCCATACCGACTATTGGGCGTCGGAGGCCGAGGGAATACAGCGGTTTCGCAAACGAGGCCAGGACTGCGTGGTCAGGAAGTGGCAGGAGCACCTCATGCGCAAGTGCCACTGTCAATCGACATTCCTTCCGACGCAGGACCGCTCGGCTCTCTGCCATTACCTGCGAGCTGCTGGCGTTTCTGCGCAGAACATCTCCGCACCTTTCGGGGCCTACAAGTTTCAACAGCTGAAAAGTTTCAACGATGTTGAAAATCAAGTTCGCACGGAGCTTTATGACCAGTTTGCGTCTGAGTGCGGCACTGTTCAGGCCTGCGAGCAGAGCAGTTACACATACTCGATCGCATCTGTTCCTTGGCCTGCGCACACCGACCTGGAGGCCTTCATCCACACGTTTGTTAGCCCGAAGTTCCACCGGGCCCGCTACCAGGGCCAGCAGCTGTTGGATCAAATCGTCAGATTTCACCGACACCCGGACGGGTCGCGGGACTTCGAGAAAGGCTGGAGCGTCAGCCACAGATTTGTGCGGCAAAACTTTGTAAAACTACAGGTCTTCGCAGAAGAGTCCAAAAGCACGCTTATTCACGAATCGCCGTCGTATTCGTTTGTTGAGGTTCTGTCTGAGCTAGGTGGAATCGGCGGCCTCTGGGTTGGCATGTCTCTGGTGACATTCGTTGAGCTGTTCGAGTTCCTGGCCATTCTGGGCATCAAGTGCACTCAAGTTGCGTCCAGCTACCTTCACTCGCGCTGGACTCGGCGAGCTGGTCAAATGGCTAACGGAGCTTGTCCGGGAAGTGCGAACCACCTGCGAACGCCTGCCTGCACCAGGTCCGCCCAATCACCCGACGAAATTACTTCGGGACCGTGCGTCGTCCTCGAGGAAGCATCCAGCGTAGTTGATGAGGACACCGAACTGGTTGGAATATTTTTATGCCCGGAAGCGTCCAGAAATGAGCACCTCCACGACGGAAATTCATCGAGACTTTCGAGAGGACAGGCAATGCAGCACGTGTCTGTTGTGGGATCC

>Macrostomum_Mlig041003.g1

GTCGACACCATGAAGAACACGCTGCATATGATGCGATGCGGCAACTTGATGGCGGCAGCAATGGCGCCTGGGCTGGGGCCTTGCGCTACGAATATTGGGCTGAACAACCTGCAGCAGCAGCAGCAACAGCAAGGAGCCCAAGAAGAGCAGCAGGGCAATCCGAACGCCCCCGGCAACGGCGGAGGCAGTCGAATACCCAGACGTCGCGAGGTGATGCTTCGCGGTTACATCAACCACTTCACCAGCGGTACGACGGCTCACGGGCTGAATCGGGTGAACGCCGAACAGAGCTGGGTTCGCATGGTCTGGGTTGGCATCGTCCTCGCAGCCTCATTTGGCGCCATCGTTCACACTACAAAGCAGGTGAAAACCTACCTGCAATATCCCGTCAGCAGTACAAATCGTCAAGAGCCGAACTCGTTCGAGTTTCCCGATTTGACATTCTGCGATCCGCTTAATAAGCGCTTCTTTCACAAGAATGTAATTAGTTACGCGCGCGAGGTAGACGTGTACGATGAGGCGCACATGATCTACGGCCTCGTCTACAGCCACTTCCGCACAGATTCGGACTGGCGCAAGGAGCGGCGCGCGATCGAACTCATCTACAACGCTCTGGAGCACTTACTGAACATTACCGACGTCAACATCCGCCCAATGGATGTTGTACTCTACTGCACATTCGACGGCGAACCGTGCAGTCACACGGATTTTCACGTGTTCTACCACCGGCTGTACACCAACTGCTTCACGTTCAAGCCGAAGCAGCGCAGCCTCCGGAGCAGCGGCATGGACCGCGGACTCTTCCTACTGCTGTACGTACCGTCAGCCGACGTCAACCTCAAACTGGACAGCTTGGCCAGGCTGGCGTCTGGTCTGGAGAACAACGGCATCCGGTTTCAGATTCACCAGCGGGACACGATCCCGCACCCGCTGGAGTACGGCATCATGGCTCCGACCGGCACCCTGACAGCGGTTGGACTCGAACAAGTGCGCACCTCCCTGGCTGACACGCCCACGAATCCGTGCGTTCGCGACACAAAACTTCGGCTATTTGATAATCAGTTCAACTTTATGCGACAGTATGACAGACAGCTGTTCGACTGTATCAAGCACCAGTACATGCGCGAAGTTCGCAAACAGTGCGGCTGCACTTTAGAGAGTCACGTGATCGCCGAGGAGGGCTTCGATCCTCGCGAAGTCGTGTTCTGTCACGATCTGATCAGCGCGAAGAACGCTACGAGAGTTGGAGCGCTCACCAAGCTCTACAATGAACTGAAGAATGTCACGAATACAGCGGGTCTTCACGGCCACATCAAGCTGGGGCTGCTCATGCGTCAGAACGACGCTGAGTTGAACGCTTCGCTGGACAGAATGATCTGCTCCGATAGCGTCTCGGAGAGTGCGGCCCGGCCGGAGCGCTGCTACGATTCGTGCAACTACAACAAATACGAGTACAGCCTCTCTCAGTGCCCTTGGCCGGAGGACTCGTTCGAGGTGCGCAACTCGCAGATCCAGATGGTGAAGATTGCGCAAATGCTGGAAGATTATAAACTGCGGTACGGTTCGGCCAATCCCGAGACCGCTCTAGCCGGACTCATGCGGAAGAGCAGTCTCAACATTAGCAGCTGTTTGACCCACGATACGCAGAAAATGCAGGTGCCAGACAAGGTTGACTGCCATCGGGTGCAGCTGTTTGTAAGAAGATCTGTTGTGCAGCTCCGCGTCTACCCGGAGACTCTGACTGTGCGTCATACGATTGAGGAGCGCAGCTACGAGCTGGTGAACCTGTGCTCAGAACTCGGCGGCATCCTGGGCCTGTGGATTGGTTTCAGCATCGTGACTCTATTCGAGTTCGCCGAATTATTCATAATATGCGCCTCATACTACTGGTACCTACTGATCAGCAAGCTGCCCAGCATCCGCAAGAGCCGGCATCCGCTTCGAATCCCAAAACCGCACCCAGTCGGCCTGCGACGCCAAATTCGCCGAGACATTGAGCTGATCTCCAACTCGGGCCAGGGGAACTCGTCATCGCAGCAGGCGTCCCCATCTCTATTGGCAACCGGCAGGCCGGCATGCACAAACAATCGAGCGCCACTGTCATCATCCTCATTTGCCAATCATCATCACCACCATCAGCTCCGGCTGGACGGTAATTCGCAGGAAACTCGGCCGCTTAGAGATGGCATGGGTGGATCC

>Phoronis_g6004.t1

GTCGACACCATGAAGAGGCAGGGTGAGATGGAGGGTCCCGATGGCTTGCTTACCAACTTCGCTCGGAGCACCTCAGCACATGGCTTAGCCCGCATCCCAGGCACCTCCCAGCCAATGCAACGCGCTGTATGGGCACTCTTGGTGACGGCCTTGGCAGCTGCTCTCATATCAGCGCTTGCAATCGTCGTTTCCGTTTACCTTCAGTACCAATACACCGAGTTCGCAAAGAAAGTGGCACGCCCTAACATCAAGTTTCCTGTCATAACAGCGTGCAACAAAGTTCCTTATGGTCTACTCCAAACAGAGCAAACTGTGAATGAGTTTTTTAGGTCAATAGGGTTGGACGAGCCTACATGTCAAAGTGAAATTTCACCAGGATTGAATGATCCCATAATATATCGTAGAATTAGCGACGCCGTTAGACCTAGAGTTCTGTACGAATACAACTCTAAAGTAATGCAAGTCTTAGGACAAAACTTGGACGACTTTATGGTGACGTGCGCCTACCAAGGACCACCATGCTTTATTTACAGTAACTTCAGCACGTCTTTATTCAAAGACCCTTACCACTATAACTGTGTAACGCTGAAAGTTCCAGACGAAGTTCAGGTAAAAGTCAACGGTCGTGGAGCAATGGAACTCGTGTTTTTCGTGGGGGACAACAAAACGTCGCTTCAAGGTAAACATGTGGACAAACTGGTTACGGATGGAACAGTTGGTATAAAATTAATAATTCATGCAGAGGGAATGCATCCCAAATGGGCTAAAACACTAGACGTGGCACCTGGCCATTTGGCCAACATCGCTGTACGACCGCGCGAAATTTACAGATTAAAAGCACCGTATGCCAGTGATTGCATAGAGAATCCACCAGATATATTGGCTGCCAGCACGAACACTTCCTATACTTACTCGAAAGAGCTGTGTACATTGAAATGTTTCAACAAACTTGTTGCGGACGCCTGCGACTGCATTCCCGAACCTGTCGTGGTGGATAAATTTGGCTTAGACTACCCGTATTGCGGAGAGTCTCCCTGCAATTATTCGTATATTTTAGATAAAATTAAATGTCACACGAACCTAACAGAGAGATTACATGAAGGTATGTTGTCTAACCAGTGTCCAGAGTGCAAGTTGCCATGTGAAGAATTGACTTATGATACTGATATGCATCTTTCAAAATGGCCTTCGAAGTTTACGTCAAAGACCATCACGAAATATATTTTAAAGAACCACGTGATTCCAAGAAACATTGAGAATGATTCTAGTGCTCTCGATTCATATGTTCAACAGAACTTTTTGAAAGTGGTGATCTACTTGGAAGACCTTCTTACAGTTGAGCAAACAGAGCAAGAGTCGATGAACTTAGCCCAGCTTACGTCTAGTGTAGGTGGCGCCATGGGTTTTTTCTTAGGGATATCAATTGTTACTGTGTTTGAGTTCATAGACCTACTAGTCAAGTCTGTACAGTCATTGTTTAAGAAAAAAAGCGTGGACAAAGTGTTGCCATTCCACAGCAAAGGATCC

>Phoronis_g5063.t1

GTCGACACCATGAACTCAGAGGTAAAAAACTGGAGTGTGAAAGACCGCATGAAAGAGTTCTGCGAGTCTACGTCAGCACACGCACTTGGGCAGACTGTAAGTTCTGGCGAAGTGAAGGCGATATTCTGGTCCCTTGTGTTCCTCAGTGCGTTAGCAGGATGTGTTTGGAATATCGTACACGTTGTGGAGTCGTACACAAGTTTTGGATTCAGCGTAAAGTCAAAACTTGAAATGGAACCGTCTTCACTGAAATTTCCAAGTGTAACTATTTGTAACCTGAACCCAATAAGTTTCTCAAGACAAGCTAGATTTGCGGGAGACTTTGAAAGGAGTACAGGTTTAGATGATGGGGATTGCGATAATGAGTTTCACAGAGATGAAACTTATCGTCCAGTCGTCTTGGATTTGTATAGGTGGTCAGAGTTACCCCAATTTTGGTTTGAATACAACGAAAATGTCACAGAAATGTTCGGCCACTCAAAGAAGGATTTTATTGTGGATTGTGTTTTTCAAAGAGAAGGTGGATGCGAAAACAACTTCTCTGTTACTAAAGACCCCAATCTGTACAACTGCTACACACTGGAACCGAATAAAAACGAAGACCTTCTTGAAATAGGATTTGCAGCTGGTTTATCGTTAACTTTATTTGTGGAAAATAGGCGTATTCGGAATGCATTTACTGGCAATTATGCGTTAGATTCCTACCACACTGGAATCGTGGGTGTCAAGGTTGCCATCCACGTGTCGGGTTCTCATCCAAATCCTAACACTCGGGGCGTGATAGCAGAAGTTGGTAAAAGCACCGACTTTATTCTGCGAACAGTAAATAGGACCGCCTTAGGTCCTCCATATCCGTCGCCCTGTAATCCCAAGAAGACAATTGAATCAAGTCATAGAAAAGAACTTCAGTATGAAGAAAATTTGTGTTTCGCAAGTTGCTTGCAAAACGCCATCGCAAAAAAATGCGGTTGTGTTTCTATCAACCCTATGGCTGTCCCTATAGCAGACAAATTCGGAATAGATCTTCCATTCTGCGGAAGTTACCCATGTAATGCGACGAAGGTGACAGAAAATTATGAATGCGTCCACGACGTAATAGGCCGTTTCTTACTTAAAAACGACCCCAATTGCCGTAACTGTACCAAGCCTTGTAACGAAGTCAGTTTTGAAGTTACAAAGTTTCAGTCAAAATGGCCGTCTGAGCTGCACCAGAGTGAGTTCATTAGGTGGCTCGCAAACAAGGACAATACAGCACTGTATAACCAGCTGGTGAGAAATATTTCAGATGACAACATTTCCAGATTTATAGAAAACAATTTTCTTCGAATCAACATATACTTTGGGGATTTTTACGTGCGCAAAGACATTGAGACTTTAGATATGGACTGGTTTGACTTGCTCTCAAGTGTTGGAGGAGCGTTTGGATTCTGGGTAGGAATTTCTGTTGTAACTGGAGTTGAAGTATTGGAGTTACTTCTTGACTGTATTGTCCTTTTTCTAAACAGGCGAATCAAAAAACGGAACCAAATAGATCTTCGAAACACTACCGGGGACGACGACGTGGTTACGGGATCC

**Other cDNA constructs.** Sources/databases described in *Experimental procedures*.

>Aplysia_FaNaC_BAE07082.1

ATGTTGGGTAGGGGTGAAAGGATAAAGCCTTACCATTTCAGGGACTCGAGCGCAGATCACATGAAATATACGAGCGTCGCGGCCAAGTCGGGAATGGTTCCTGAGCACCGGTACACGATGGTGAGGAGCCGGCACCACGGCCGCCACCACCACCACCACAGCTACCAGGAGTACAACACGCAGCGCTCCGCCATCAGCCTGATCGCCGAGCTGGGCTCGGAGAGCAACGCCCACGGCTTGGCCAAGATCGTCACGTCCCGCGACACCAAGCGGAAGGTCATCTGGGCGCTGATGGTCATCATCGGTTTCACGGCCGCCACGCTGCAGCTTTCACTCCTGGTGCGGAAGTACCTGCAGTTCCAGGTGGTGGAACTGTCCGAGATCAAAGACAGCATGCCCGTGGAGTACCCGTCCGTGACCATCTGCAACATCGAGCCCATCTCGCTGAGGAAGATTCGGAAGGCGTACAATAAGAACGAGAGTCAGAACTTGAAAGACTGGCTCAACTTCACGCAGACGTTTCACTTCAAGGATATGTCTTTCATGAACAGCATCCGCGCGTTCTACGAGAATTTGGGCACGGACGCCAAGAAGATCAGCCATGACCTCCGTGACCTCCTCATCCACTGCCGGTTCAATCGAGAAGAGTGCACCACGGAGAACTTCACGTCCTCCTTCGACGGGAACTACTTCAACTGCTTCACTTTCAACGGCGGCCAGTTACGGGATCAGCTACAGATGCACGCCACAGGTCCGGAAAACGGGCTCTCGCTCATTATCTCTATAGAGAAAGATGAACCGCTTCCCGGGACGTATGGAGTATACAATTTCGAGAACAACATCCTACACAGCGCCGGCGTACGTGTCGTGGTGCACGCCCCGGGTTCGATGCCCAGCCCGGTGGACCACGGCTTCGACATCCCGCCCGGGTACTCATCCTCCGTGGGTCTGAAAGCTCTGCTCCACACGCGCCTTTCTGAGCCGTACGGCAACTGCACCGAGGACTCACTCGAGGGAATCCAGACGTACCGCAACACGTTCTTCGCCTGCCTGCAGCTGTGTAAACAAAGGAGGCTCATTAGGGAGTGTAAGTGTAAGTCCTCGGCCCTCCCAGATTTAAGTGTGGAGAACATCACGTTCTGCGGAGTCATTCCGGACTGGAAGGATATACGGAGAAACGTCACTGGAGAATACAAGATGAACCAGACAATCCCCACCATCTCCTTGGCGTGTGAAGCGCGCGTGCAGAAGCAGCTCAACAACGACCGCTCCTACGAGACGGACTGCGGTTGCTACCAGCCTTGTAGCGAGACGTCATACCTCAAGTCGGTCTCCCTCTCATACTGGCCCCTAGAGTTTTATCAGCTCAGCGCGTTAGAGAGATTCTTCAGCCAGAAGCACCCGACGGACCAGCAGCACTTCATGAAGATCGCCCAAGACTTTCTGTCCCGCCTGGCGCACCCACAGCAGCAGGCACTGGCCCGCAACAACAGCCACGACAAAGACATCCTCACCACCAGCTACTCCCTCTCCGAGAAAGAGATGGCGAAAGAGGCCTCGGATCTAATACGCCAGAACCTTCTCAGGCTCAATATATACCTAGAGGACCTGAGCGTGGTGGAGTACCGCCAGCTCCCCGCCTACGGGCTGGCCGACCTGTTCGCGGACATCGGCGGCACGCTGGGCCTGTGGATGGGCATCTCCGTGCTCACCATCATGGAGCTCATGGAGCTCATCATCCGCCTCTTCGCGCTCATCTTCAACGCCGAGCGGGAGGTGCCCAAAGCGCCCATGCACAACAGCAACAACGGCGGAAGCGGCGGCGGCGACGGTAGCGGTGGACAGCACAACTTCGCCAACGGGGACGTGGAGCATGAGCGGGACACGCACTTCCCCGACCTCGGCTCCAGCGATTTCGATTTTCGCCGCGGCGGCGGGATAGGCGCGGAGTCGCCCGTATG

>Rat_ASIC1a_NP_077068.1

ATGGAATTGAAGACCGAGGAGGAGGAGGTGGGTGGTGTCCAGCCGGTGAGCATCCAGGCTTTCGCCAGCAGCTCCACGCTGCATGGTCTTGCCCACATCTTCTCCTATGAGCGGCTGTCTCTGAAGCGGGCACTGTGGGCCCTgtGCTTCCTGGGTTCGCTGGCCGTCCTGCTGTGTGTGTGCACTGAGCGTGTGCAGTACTACTTCTGCTATCACCACGTCACCAAGCTTGACGAAGTGGCTGCCTCCCAGCTCACCTTCCCTGCTGTCACACTGTGCAATCTCAATGAGTTCCGCTTTAGCCAAGTCTCCAAGAATGACCTGTACCATGCTGGGGAGCTGCTGGCCCTGCTCAACAACAGGTATGAGATCCCGGACACACAGATGGCTGATGAAAAGCAGCTAGAGATATTGCAGGACAAGGCCAACTTCCGGAGCTTCAAGCCCAAGCCCTTCAACATGCGTGAATTCTACGACAGAGCGGGGCACGATATTCGAGACATGCTGCTCTCGTGCCACTTCCGTGGGGAGGCCTGCAGCGCTGAAGATTTCAAAGTGGTCTTCACTCGGTATGGGAAGTGTTACACATTCAACTCGGGCCAAGATGGGCGGCCACGGCTGAAGACCATGAAAGGTGGGACTGGCAATGGCCTGGAGATCATGCTGGACATTCAGCAAGATGAATATTTGCCTGTGTGGgGAGAGACCGACGAGACATCCTTCGAAGCAGGCATCAAAGTGCAGATCCACAGTCAGGATGAACCCCCTTTCATCGACCAGCTGGGCTTTGGTGTGGCTCCAGGTTTCCAGACGTTTGTGTCTTGCCAGGAGCAGAGGCTCATCTACCTGCCCTCACCCTGgGGCACCTGCAATGCTGTTACCATGGACTCGGATTTCTTCGACTCCTACAGCATCACTGCCTGCCGGATTGATTGCGAGACGCGtTACCTGgtGGAGAACTGCAACTGCCGTATGGTGCACATGCCAGGGgaCGCCCCATACTGCACTCCAGAGCAGTACAAGGAGTGTGCAGATCCTGCCCTGGACTTCCTAGTGGAGAAAGACCAGGAATACTGCGTGTGTGAGATGCCTTGCAACCTGACCCGCTACGGCAAGGAGCTGTCCATGGTCAAGATCCCAAGCAAAGCCTCCGCCAAGTACCTGGCCAAGAAGTTCAACAAATCGGAGCAGTACATAGGGGAGAACATTCTGGTGCTGGACATTTTCTTTGAAGTCCTCAACTATGAGACCATCGAGCAGAAAAAGGCCTATGAGATCGCAGGGCTGTTGGGTGACATCGGGGGCCAGATGGGGTTGTTCATCGGTGCCAGCATCCTCACCGTGCTGGAACTCTTTGACTATGCCTACGAGGTCATTAAGCACAGGCTGTGCAGACGTGGAAAGTGCCAGAAGGAGGCTAAGAGGAGCAGCGCAGACAAGGGCGTGGCGCTCAGCCTGGATGACGTCAAAAGACACAATCCCTGCGAGAGCCTCCGAGGACATCCTGCCGGGATGACGTACGCTGCCAACATCCTACCTCACCATCCCGCTCGAGGCACGTTTGAGGACTTTACCTGCTAA

**Supporting references**

42. Kumar, S., Tumu, S. C., Helm, C., and Hausen, H. (2020) The development of early pioneer neurons in the annelid Malacoceros fuliginosus. *BMC Evol. Biol.* **20**, 117

45. Krieg, P. A., and Melton, D. A. (1984) Functional messenger RNAs are produced by SP6 in vitro transcription of cloned cDNAs. *Nucleic Acids Res.* **12**, 7057-7070
